# Modeling the Effect of APC Truncation on Destruction Complex Function in Colorectal Cancer Cells

**DOI:** 10.1371/journal.pcbi.1003217

**Published:** 2013-09-26

**Authors:** Dipak Barua, William S. Hlavacek

**Affiliations:** 1 Theoretical Biology and Biophysics Group, Theoretical Division and Center for Nonlinear Studies, Los Alamos National Laboratory, Los Alamos, New Mexico, United States of America; 2 Department of Biology, University of New Mexico, Albuquerque, New Mexico, United States of America; 3 Clinical Translational Research Division, Translational Genomics Research Institute, Phoenix, Arizona, United States of America; Princeton University, United States of America

## Abstract

In colorectal cancer cells, APC, a tumor suppressor protein, is commonly expressed in truncated form. Truncation of APC is believed to disrupt degradation of β—catenin, which is regulated by a multiprotein complex called the destruction complex. The destruction complex comprises APC, Axin, β—catenin, serine/threonine kinases, and other proteins. The kinases 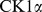 and 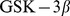, which are recruited by Axin, mediate phosphorylation of β—catenin, which initiates its ubiquitination and proteosomal degradation. The mechanism of regulation of β—catenin degradation by the destruction complex and the role of truncation of APC in colorectal cancer are not entirely understood. Through formulation and analysis of a rule-based computational model, we investigated the regulation of β—catenin phosphorylation and degradation by APC and the effect of APC truncation on function of the destruction complex. The model integrates available mechanistic knowledge about site-specific interactions and phosphorylation of destruction complex components and is consistent with an array of published data. We find that the phosphorylated truncated form of APC can outcompete Axin for binding to β—catenin, provided that Axin is limiting, and thereby sequester β—catenin away from Axin and the Axin-recruited kinases 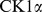 and 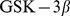. Full-length APC also competes with Axin for binding to β—catenin; however, full-length APC is able, through its SAMP repeats, which bind Axin and which are missing in truncated oncogenic forms of APC, to bring β—catenin into indirect association with Axin and Axin-recruited kinases. Because our model indicates that the positive effects of truncated APC on β—catenin levels depend on phosphorylation of APC, at the first 20-amino acid repeat, and because phosphorylation of this site is mediated by 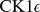, we suggest that 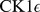 is a potential target for therapeutic intervention in colorectal cancer. Specific inhibition of 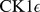 is predicted to limit binding of β—catenin to truncated APC and thereby to reverse the effect of APC truncation.

## Author Summary

We asked the question, how can the effects of APC truncation, a very common mutation in colorectal cancer, be understood and reversed? We addressed this question by formulating a computational model for destruction complex function that incorporates site-specific details about protein-protein interactions and protein phosphorylation and examined the differences in predicted behaviors when APC is full length, as in normal cells, and truncated, as in colorectal cancer cells. Our model offers an explanation for how and why destruction complex function is altered by APC truncation. The model indicates that phosphorylation of the first 20-amino acid repeat in APC (which is usually the only 20-amino acid repeat that remains in truncated forms of APC) together with the absence of SAMP repeats (missing entirely because of truncation) allows truncated APC to act as a diversion sink. In other words, phosphorylated APC can outcompete Axin for binding to , provided Axin is limiting, and thereby prevent  from associating with Axin and the Axin-associated kinases  and , which initiate phosphorylation-dependent degradation of . Thus, the model identifies inhibition of APC phosphorylation, which is mediated by , as a potential means by which the oncogenic effect of APC truncation could be reversed.

## Introduction


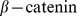
 (CTNNB1) is a key signaling protein in the 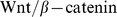 pathway [1], [2], a regulator of cadherin cell adhesion molecules [3], and a regulator of the Tcf and Lef family of transcription factors [4]–[7]. In mesenchymal cells, 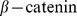 levels increase when a Wnt ligand binds a cell-surface Frizzled (Fz)-family receptor. Activation of the Wnt/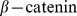 pathway (transiently) inhibits proteosome-mediated degradation of 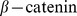. Wnt binding also has other important effects on 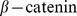, including regulation of phosphorylation state and redistribution of 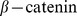 within subcellular compartments. In colorectal cancer cells, normal control of 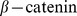 degradation is disrupted, resulting in elevated levels of 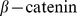.

Cellular degradation of 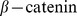 is regulated by (in our view) oligomeric protein complexes, which have diverse compositions but common features; these complexes are often collectively referred to as the 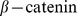 destruction complex [8]–[10]. The destruction complex, which characteristically contains 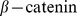 and two scaffold proteins, Axin (axis inhibition protein, AXIN1) and APC (adenomatous polyposis coli protein), mediates phosphorylation of 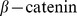 by recruiting 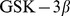 (glycogen synthetase 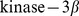, GSK3B) and 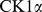 (casein kinase 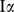, CSNK1A1) [11]–[15]. These kinases, upon binding Axin, catalyze phosphorylation of 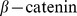 on specific serine and threonine residues. Phosphorylation of Ser-45 by 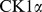 and subsequent phosphorylation of Ser-33, Ser-37, and Thr-41 by 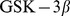 initiates ubiquitination and proteosome-mediated degradation of 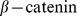 [12]–[15]. The destruction complex also recruits PP2A, a multimeric protein phosphatase, which opposes the action of kinases. It has been suggested that activation of Wnt/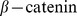 signaling destabilizes the destruction complex by sequestering Axin in complexes with activated Fz receptors [16]–[18]. However, details about the early events in Wnt/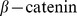 signaling are still emerging [19], [20]. In colorectal cancer cells, the destruction complex member APC is often truncated [21]. An important effect of APC truncation is believed to be a perturbation of the interactions amongst proteins comprising the destruction complex that alters regulation of 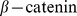 degradation, perhaps by destabilizing the destruction complex in a way similar to the destabilization brought about by Wnt signaling.

The interactions responsible for assembly of the destruction complex are complex and are mediated by multiple functional sites within the member proteins of the destruction complex. The characteristic core of the destruction complex can be viewed as a ternary complex that forms through interactions of APC, Axin, and 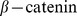. 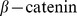 contains twelve ARM (Armadillo) repeats, allowing it to bind both APC and Axin. In particular, ARM repeats 3 and 4 constitutively bind a central region of Axin [22], [23] as well as a phosphorylated 20-amino acid (20-aa) repeat region of APC [24], [25]. There are total of seven 20-aa repeats in this region. 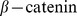 ARM repeats 5–9 constitutively bind three 15-amino acid (15-aa) repeats in the N-terminal region of APC [26]. APC contains three SAMP (serine-alanine-methionine-proline) repeats, which bind the RGS (regulator of G protein signaling) domain of Axin [27]. These interactions connect the three core proteins of the destruction complex (APC, Axin, and 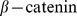) and enable each protein to bind the other two core proteins, possibly within a closed/cyclic ternary complex. A cyclic complex would presumably be highly stable, because dissociation of such a complex would require the sequential break up of two protein-protein interactions.

Stability of the destruction complex may be important for its function as a platform for phosphorylation of 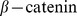, and other proteins. The destruction complex mediates phosphorylation of 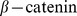 by allowing Axin to colocalize the kinases 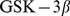 and 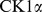 with their substrate 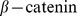. Axin contains binding sites for both 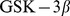 [11], [28] and 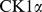 [12], [29]. Interestingly, the destruction complex is also thought to mediate phosphorylation of APC by colocalizing another kinase, 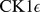 (CSNK1E), with APC [30], although it is not known which protein in the destruction complex recruits 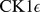. 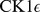 and 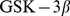 together mediate phosphorylation at the 20-aa repeat region of APC [30]. Recall that this region in APC, when phosphorylated, mediates interaction with a site in 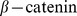 that also interacts with Axin [22]–[25]. Thus, phosphorylated APC and Axin compete for binding to 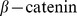. The outcome of this competition is perhaps dependent on stability of the destruction complex.

Much of what we know about the functional effects of APC truncation has come from studies of a human colon adenocarcinoma cell line (SW480). SW480 cells express a truncated form of APC termed APC1338, which contains only the first 1,338 amino acids of the full-length protein [9], [31]. APC1338 contains all three 15-aa repeats and the first 20-aa repeat, but is devoid of the remaining six 20-aa repeats and the SAMP repeats, which bind Axin [9], [31]. Therefore, a model can be conceptualized wherein assembly of the functional destruction complex cannot be completed in the absence of interaction between APC1338 and Axin, leading to decreased phosphorylation, ubiquitination, and degradation of 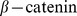 [2]. However, an absence of SAMP repeats in APC does not prevent direct binding of Axin to 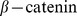 [22], [23], and there are some uncertainties about the validity of this model [19] because reports from different laboratories have shown that expression of recombinant APC can either promote degradation of 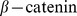 or have no or little effect, depending on cell type and whether APC is expressed transiently or stably [31]–[34].

As discussed above, APC plays an important role in destruction complex function. However, APC is a multifunctional protein, subject to numerous post-translational modifications. It is believed to play a role in regulating not only phosphorylation and ubiquitination of 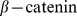 but also localization of 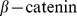. There are several pools of 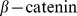: membrane-associated (e.g., complexed with E-cadherin), cytosolic (free, 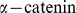 bound and Tcf bound), and nuclear. Other components of the destruction complex are also multifunctional proteins, which can be found in distinct subcellular locations and states. For example, Axin, through self-polymerization mediated by its DIX (dishevelled and axin) domain [35], localizes to cytoplasmic puncta. We will not consider these complexities, but they are mentioned at this point to caution the reader about the limitations of our study.

Here, our focus will be on APC regulation of 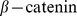 phosphorylation within an idealized destruction complex, taken to comprise a ternary complex of APC, Axin, and 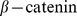 with 1∶1∶1 stoichiometry. We will consider the site-specific details of the interactions amongst these proteins, because these details are relevant for understanding how the interactions of APC, Axin, and 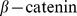 are perturbed by an absence of SAMP repeats in truncated APC (APC1338). We also consider, with less mechanistic resolution, proteins that mediate phosphorylation and dephosphorylation of APC and 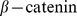 and degradation of 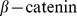. The set of proteins of interest are considered in isolation. Thus, for example, we do not consider 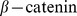 interaction with E-cadherin, or the effects of Wnt. We also do not consider Axin puncta or the DIX domain in Axin. Axin puncta play a role in 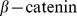 degradation but are not required for phosphorylation of 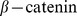 [36].

To investigate the roles of APC and its oncogenic truncated forms in destruction complex function, we formulated a computational model for regulation of 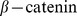 phosphorylation and degradation using local rules to represent the protein-protein interactions of interest [37]–[39]. This rule-based approach, ideal for modeling the chemical kinetics of biomolecular interaction networks, allowed us to consider the mechanistic details of protein-protein interactions at the resolution level of functional sites within the proteins of interest. These mechanistic details are complex, as summarized above, and arguably beyond our ability to comprehend without reasoning aids, such the model considered here. Using this model, we interrogated system behavior, which emerges from the states and state changes of protein sites, with the goal of elucidating the distinctive mechanisms by which APC and APC1338 regulate the rate of 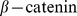 destruction in normal and SW480 colorectal cancer cells. We also used our model to investigate the functional significance of intracomplex interactions among APC, Axin, and 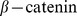, which have the potential to produce a highly stable cyclic ternary complex.

Although APC is a characteristic component of the destruction complex and thought to be important for degradation of 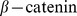 [31]–[34], our analyses suggest that APC does not promote degradation of 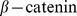 in a normal cell when overexpressed. However, we do predict that expression of recombinant full-length APC in SW480 cells promotes 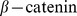 degradation, as seen in several studies [31]–[34]. These results are obtained because, according to our model, phosphorylated APC1338 in SW480 cells competes with Axin for 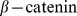. APC1338-mediated separation of 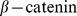 from Axin reduces phosphorylation of 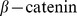 by Axin-recruited kinases, and reduced phosphorylation of 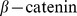 decreases its rate of degradation. In contrast, in normal cells, binding of phosphorylated full-length APC to 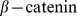, in competition with Axin, is not functionally equivalent because Axin can still colocalize with 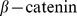 through indirect association via the SAMP repeats in APC, which are missing in APC1338. Because of these results and because 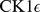 is responsible for phosphorylation of APC (but not 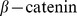), we identify 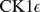 as a potential target for therapeutic intervention in colorectal cancer. Inhibition of 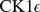 is predicted to limit sequestration of 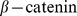 away from Axin and Axin-associated kinases and thereby to lower 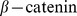 levels in cancer cells expressing truncated APC.

## Results

To investigate how the function of the 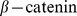 destruction complex changes when APC is mutated, especially as a result of a typical C-terminal truncation that removes the SAMP repeats and all but the first of the 20-aa repeats, we formulated a model (as described below) for full-length APC interactions with other components of the destruction complex. We then used this model and variants corresponding to different mutated forms of APC to predict how 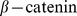 levels and other readouts of system behavior depend on various parameters, such as the abundance of APC or truncated APC. Because APC contains multiple functional components or sites and we are interested in forms of APC containing different subsets of these sites, we formulated a model that tracks the chemical kinetics of the protein-protein interactions of interest with site-specific/structural resolution. This was accomplished by leveraging the rule-based modeling approach [37], [40], in which local rules are used to represent biomolecular interactions and their consequences. Modeling with site-specific resolution is difficult with traditional modeling approaches, such as that of ordinary differential equations (ODEs), because of combinatorial complexity [41], which arises from multisite phosphorylation, multivalent binding, and other common aspects of biomolecular interactions involved in cellular regulation. Combinatorial complexity is a motivating factor for the use of rule-based modeling here.

### Model

**Figure 1:**
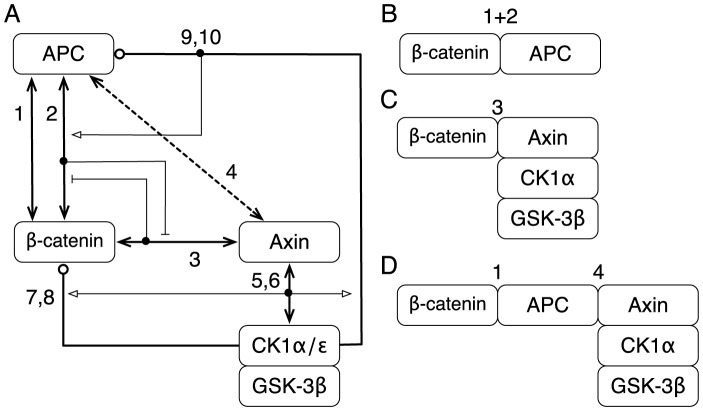
Overview of the signaling proteins and interactions considered in the model. Panel A is a simplified version of Fig. 2, which follows and goes beyond the diagram shown here by illustrating the functional components of proteins responsible for interactions. Selected protein complexes considered in the model are illustrated in Panels B–D. (A) Proteins are represented by boxes. In the model, five proteins, 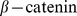, APC, Axin, 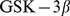, and 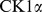, are considered explicitly, whereas 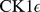, PP2A (not shown), and other proteins are considered implicitly. 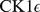, which mediates phosphorylation of APC, and PP2A, which mediates dephosphorylation of APC, are assumed to be constitutively associated with Axin. In the model, their activities are engaged when Axin is in complex with APC. Interactions included in the model are represented by arrows; numbering of arrows is the same as in Fig. 2. The arrows labeled 1–6 represent reversible direct binding interactions. The arrows labeled 7–10 represent catalytic (phosphorylation) interactions (and enzyme-substrate relationships). All phosphorylation events are taken to be reversed by phosphatases. The interaction represented by Arrow 1 is constitutive. The interaction represented by Arrow 2 depends on sequential phosphorylation of APC by 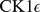 and 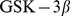 (Arrows 9 and 10). The interactions represented by Arrows 2 and 3 are mutually exclusive, because they involve the same binding site in 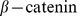, i.e., Axin and APC compete for binding to this site. The interaction between APC and Axin represented by Arrow 4 is missing for typical truncated forms of APC (i.e., forms of APC, such as APC1338, missing SAMP repeats). Arrows 5 and 6 represent recruitment of 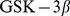 and 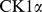 to Axin. Arrows 7–10 represent phosphorylation reactions mediated by Axin-associated kinases. (B) A binary complex of APC and 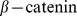 connected through two distinct protein-protein interfaces. The interactions represented by Arrows 1 and 2 are allowed to occur simultaneously. (C) A complex wherein 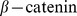 is directly bound to Axin via the interaction represented by Arrow 3. Recall that this interaction cannot occur if 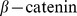 is bound to APC via the interaction represented by Arrow 2. (D) A complex containing a linear (vs. cyclic) ternary complex of APC, Axin, and 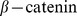. This linear complex is allowed to close and form a cyclic complex via the interaction represented by Arrow 3. Note that the complex depicted here cannot form when APC is truncated such that the interaction represented by Arrow 4 is missing. The model is further described in Fig. 2 and Text S1.

**Figure 2:**
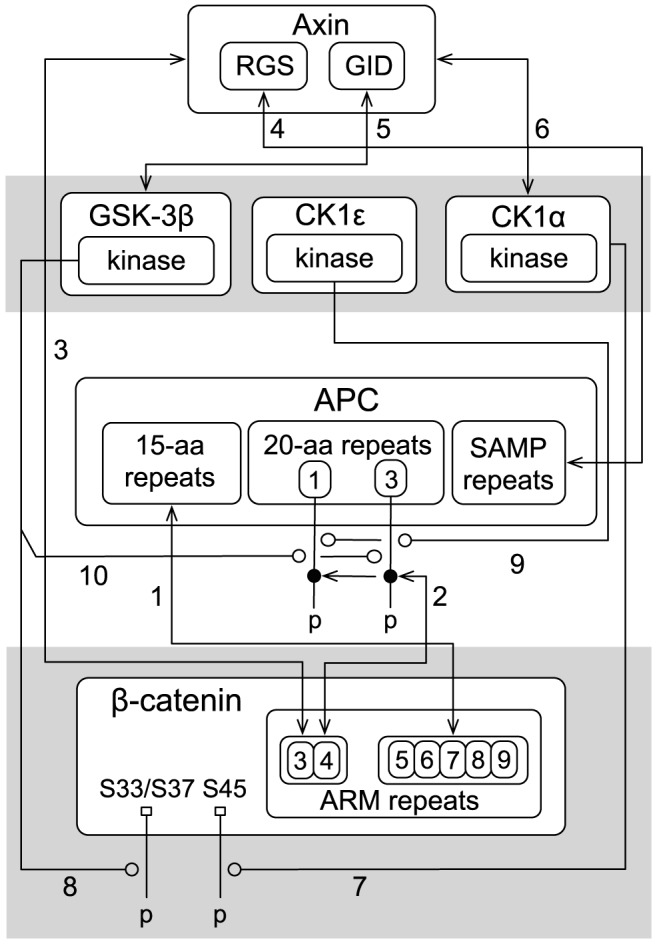
Site-specific details of the proteins and interactions considered in the model. Proteins, interactions, and the functional components that mediate interactions are represented according to the conventions of Chylek et al. [42]. The numbering of arrows is the same as in Fig. 1. The double-arrowed lines represent reversible binding interactions. The lines ending with an open circle represent enzyme-substrate relationships and point to sites of phosphorylation. In the model, 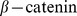 sites Ser-33, Ser-37, and Thr-41, which are 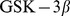 substrates, are lumped together as a single site labeled S33/37. 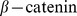 site Ser-45, which is a 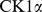 substrate, is labeled S45. In the model, the seven 20-aa repeats of APC are lumped into two distinct sites labeled 1 and 3. For further information about the model, see Materials and Methods. A complete and executable specification of the model is provided in the Supporting Information as a plain-text BioNetGen input file (Text S2). Note that there is a correspondence between the arrows shown here and the rules of the model (Text S1). Model parameter values are summarized in Table 1.

We developed a model for APC, Axin, and 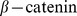 interactions and destruction complex function using the rule-based modeling framework of BioNetGen [37]–[39] (see Materials and Methods). We considered a base model, corresponding to a normal cell with full-length APC, and several variant forms of the base model. The base model is illustrated in Figs. 1 and 2. The model is annotated in Text S1 (Supporting Information). Executable BioNetGen input files are provided in the Supporting Information for the base model (Text S2) and eight variant forms of the base model (Text S3 through Text S10).

In the base model, both explicit and implicit interactions are considered. We explicitly consider the interactions of five signaling proteins (and their isoforms presumed to be functionally equivalent): APC, Axin, 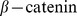, 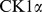, and 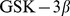. We implicitly consider the interactions of 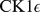, PP2A, other phosphatases, and the proteins responsible for ubiquitination and proteosomal degradation of 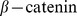. In Fig. 2, proteins and their interactions are represented with site-specific/structural resolution using the conventions of Chylek et al. [42]. Briefly, proteins and their functional components are represented by nested boxes. Components excluded from consideration (e.g., the DIX domain of Axin) are not illustrated in Fig. 2. Arrows connecting boxes represent interactions. It should be noted that the visual elements of Fig. 2 correspond to the formal elements of our model [42]: boxes correspond to molecule types and arrows correspond to rules for interactions (Text S1). Each interaction included in the model is discussed below. The technical details of how these interactions are modeled/represented using rules are explained in Text S1. See also the Materials and Methods section.

Arrow 1 in Fig. 2 represents reversible binding of 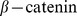 ARM repeats 5–9 to the 15-aa repeats of APC [22], [26]. In the model, ARM repeats 5–9 are considered to comprise a single binding site. Likewise, the three 15-aa repeats in APC are considered to comprise a single binding site.

Arrow 2 represents reversible binding of 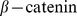 ARM repeats 3 and 4 to phosphorylated APC 20-aa repeats [22]. In the model, ARM repeats 3 and 4 are considered to comprise a single binding site. The seven 20-aa repeats of APC are taken to function as two distinct binding sites, with binding activity of one site considered to be mutually exclusive with binding activity of the other site. The first site (labeled 1) corresponds to the first 20-aa repeat and the second site (labeled 3) corresponds to the third 20-aa repeat. We consider binding of APC to 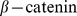 to be mediated by the phosphorylated first repeat when the protein is APC1338 (or a comparable truncated form of APC), and predominantly (exclusively in the model as a simplification) by the phosphorylated third repeat if the protein is full-length APC. This distinction is made because APC1338 contains only the first 20-aa repeat, whereas full-length APC contains all seven 20-aa repeats. Binding of full-length APC to 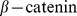 is mediated primarily by the phosphorylated third 20-aa repeat [25] because the phosphorylated third repeat binds with 100- to 1000-fold higher affinity than that of any of the other phosphorylated 20-aa repeats [25]. We take the stoichiometry of a 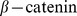-APC complex to be 1∶1.

Arrow 3 represents reversible binding of 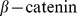 to Axin. ARM repeats 3 and 4 of 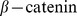 bind a central region of Axin [22], [23]. As noted before, ARM repeats 3 and 4 also bind the phosphorylated 20-aa repeat region of APC (Arrow 2). Thus, ARM repeats 3 and 4 represent a 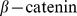 binding site recognized by both APC and Axin.

Arrow 4 represents reversible binding of APC to Axin. The three SAMP repeats of APC bind the RGS domain of Axin [27], [43]. In the model, as a simplification, the SAMP repeats are considered to comprise a single binding site. Thus, we take the stoichiometry of an APC-Axin complex to be 1∶1.

Arrows 5 and 6 represent reversible binding of 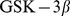 and 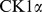 to Axin, respectively. 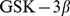 binds the GSK3 interaction domain (GID) of Axin [11], [28], [44]. 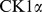 binds a central region of Axin [29], which is distinct from the binding sites in Axin recognized by other binding partners. In the model, the binding of 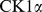, 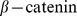 and 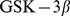 to Axin is taken to be non-competitive and non-cooperative.

Arrows 7 and 8 represent phosphorylation of 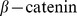 by Axin-bound 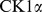 and 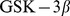, respectively. 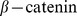 phosphorylation takes place in a processive manner [12], [13]. 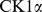 first phosphorylates Ser-45 (labeled as S45 in Fig. 1), and 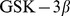 then phosphorylates Ser-33, Ser-37, and Thr-41. In the model, as a simplification, the latter three sites are lumped together (labeled as S33/S37 in Fig. 2). We model the phosphorylation reactions as processes with first-order kinetics that occur only when kinases and substrates are colocalized within a complex. In the model, phosphorylation at S45 occurs when 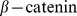 is colocalized with Axin-associated 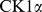. Phosphorylation at S33/S37 occurs when 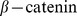 is phosphorylated at S45 and colocalized with Axin-associated 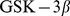 [12], [13]. We do not consider phosphorylation of 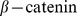 outside the context of the destruction complex.

Arrows 9 and 10 represent phosphorylation of APC 20-aa repeats by 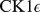 and 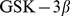 [30], [45]. Both 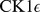 and 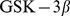 are required for phosphorylation of APC [30]. In Fig. 2, 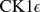 is shown for illustration purposes only. In the model, we implicitly consider 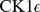 because it is not known which protein is responsible for colocalizing 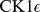 with APC. Phosphorylation of APC is taken to occur through a process with first-order kinetics when APC and 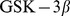 are colocalized via Axin. Thus, we assume that 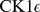 is colocalized with APC in proportion to the extent to which 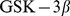 is colocalized with APC via Axin. This assumption is equivalent to assuming that 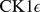 associates non-competitively with Axin (or directly with 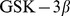).

We model dephosphorylation reactions as first-order processes (without explicit consideration of phosphatases). We allow dephosphorylation to occur if a site is exposed, i.e., not occupied and shielded by a binding partner. In the model, both phosphorylation sites of 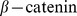 (i.e., S45 and S33/S37) are dephosphorylated according to the same rate law. In other words, the same first-order dephosphorylation rate constant is used for both sites. We allow the 20-aa repeats in APC to be dephosphorylated only if APC is in complex with Axin because Axin recruits PP2A, a phosphatase that mediates dephosphorylation of APC [46].

An important feature of the model is intracomplex binding of APC, Axin, and 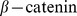. In Fig. 2, Arrows 1–4 each represents two distinct types of binding reactions: intermolecular binding, and intracomplex binding. The former type of binding reaction occurs when the reacting sites are freely diffusing, i.e., not tethered. The latter type of binding reaction occurs when the reacting sites are already in a complex together, i.e., tethered and co-confined to a small subvolume of the cytoplasm. An intracomplex reaction can be marked by a high apparent affinity because of the high local concentrations of the tethered binding partners [47]. In the model, these reactions lead to complex stabilization. We account for the high local concentration effect on an intracomplex reaction by multiplying the corresponding forward rate constant by an enhancement factor 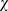. For instance, if 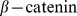 and APC are already connected via Axin, then the effective forward rate constant for the reaction represented by Arrow 1 would be 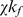, where 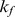 is the intrinsic forward rate constant when the proteins are not tethered together.

It should be noted that in the model 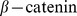 and APC can form a binary complex held together by two-point attachment i.e., 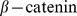 and APC can be held together through simultaneous interaction between 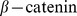 ARM repeats 3 and 4 and APC 20-aa repeats (Arrow 1) and interaction between 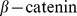 ARM repeats 5–9 and APC 15-aa repeats (Arrow 2). The intracomplex reactions between APC and 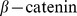 are allowed to occur outside the context of a completely assembled destruction complex.

In the model, except for 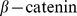, the total concentrations of signaling proteins are taken to be conserved (i.e., constant). 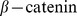 is produced in a process with zeroth-order kinetics and degraded in either a slow or fast process with first-order kinetics. When S33/S37 is not phosphorylated, 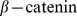 is degraded at a slow rate, regardless of its bound state. When S33/S37 is phosphorylated, 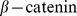 is degraded at a fast rate, again regardless of its bound state. Thus, we allow 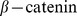 to be degraded, through a slow or fast process, independently of whether it is free or bound. We assume that 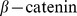 releases any binding partner(s) upon degradation.

The model has 27 independent parameters, including five protein concentrations and 14 binding constants (Table 1). Parameter values were specified as described in Materials and Methods. A local sensitivity analysis indicates that model behavior is not particularly sensitive to any individual parameter value (Table S1).

**Table 1:** Model parameter 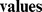 1.

Parameters	Comments
*BCAT_tot_* = 35 nM (1.1×10^4^ copies/cell)	*β*–catenin concentration [53], [59]
*APC_tot_* = 100 nM (3.2×10^4^ copies/cell)	APC concentration [53], [59]
*AXIN_tot_* = 10 nM (3.2×10^3^ copies/cell)	Axin concentration [59]
*GSK_tot_* = 100 nM (3.2×10^4^ copies/cell)	GSK–3*β* concentration [53], [59]
*CKl1α_tot_* = 100 nM (3.2×10^4^copies/cell)	CK1*α* concentration (assumed)
*β*–catenin ARM repeats 5–9 binding to the APC 15-aa repeat region	
*K_D_*_1,*bap*_ = 273 nM	Equilibrium dissociation constant [43]
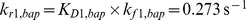	Dissociation rate constant^2^
*β*–catenin ARM repeats 3 and 4 binding to the phosphorylated APC 20-aa repeat	
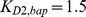 nM	Equilibrium dissociation constant [25]
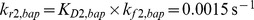	Dissociation rate constant^2^
*β*–catenin ARM repeats 3 and 4 binding to the phosphorylated APC1338 20-aa repeat	
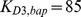 nM	Equilibrium dissociation constant [25]
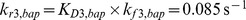	Dissociation rate constant^2^
*β*–catenin ARM repeats 3 and 4 binding to Axin	
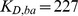 nM	Equilibrium dissociation constant [43]
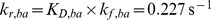	Dissociation rate constant^2^
Axin binding to the APC SAMP repeats	
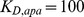 nM	Equilibrium dissociation constant (assumed)
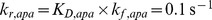	Dissociation rate constant^2^
GSK–3*β* binding to Axin	
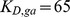 nM	Equilibrium dissociation constant [68]
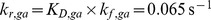	Dissociation rate constant^2^
CK1*α* binding to Axin	
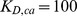 nM	Equilibrium dissociation constant (assumed)
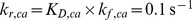	Dissociation rate constant^2^
*β*–catenin phosphorylation and dephosphorylation (at both S33/S37 and S45 sites)	
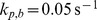	*β*–catenin phosphorylation rate constant
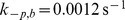	*β*–catenin dephosphorylation rate constant^3^
APC/APC1338 phosphorylation and dephosphorylation at the 20-aa repeat region (site 1 or 3)	
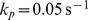	APC phosphorylation rate constant^4^
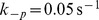	APC dephosphorylation rate constant^4^
*β*–catenin synthesis and degradation	
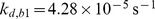	Slow degradation rate constant^4^
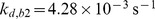	Fast degradation rate constant^4^
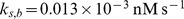 (4.0 molecules/s)	Synthesis rate constant^4^
Enhancement factor	
*χ* = 10^4^ nM	Enhancement factor for intracomplex binding^4^

1 Unit conversions are based on a cell cytoplasmic volume of 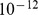 L [59].

2 For each binding reaction, the association rate constant (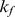) is assumed to be 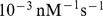.

3 The half-life 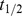 of 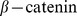 phosphorylation is approximately 10 min [74].

4 The selected parameter values allow the model to reproduce a number of experimental observations, including 1) the steady-state 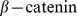 level, 2) the half-lives of 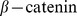 and S33/S37-mutated 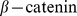 (Fig. S1), and 3) the kinetics of dephosphorylation of 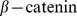 at S33/S37 and S45 upon treatment with LiCl (Fig. S2).

See Materials and Methods for more details.

### Effects of APC mutation on *β*–catenin expression

Using the estimated parameter values summarized in Table 1 (see Materials and Methods), which were selected in part to allow the model to reproduce certain system behaviors (Figs. S1 and S2), we tested whether the model is able to predict the effects of transfection of SW480 cells with different truncated forms of APC. Munemitsu et al. [31] systematically transfected SW480 cells with various forms of APC. These experiments were designed to understand the effects of deletion of different functional components of APC on 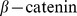 levels in SW480 cells, which almost exclusively express APC1338 instead of the full-length protein [31], [48].

**Figure 3:**
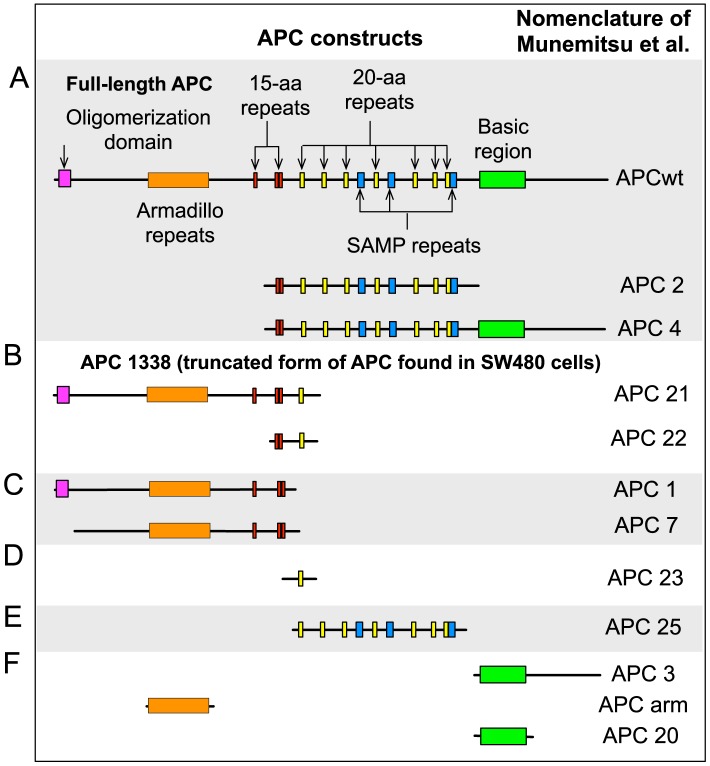
Summary of APC constructs considered in simulated transfections and in the experimental study of Munemitsu et al. [31]. The 12 constructs used by Munemitsu et al. [31] are divided into six classes based on their structures. Proteins within the same class are functionally equivalent according to our model. A representative of Class A (APC-A) contains all three protein binding sites considered in the model for full-length APC. This class is regarded as equivalent to full-length APC. A representative of Class B (APC-B) contains 15-aa repeats and the first 20-aa repeat. This class is regarded as equivalent to APC1338, the truncated form of APC found in SW480 cells. A representative of Class C (APC-C) corresponds to a fragment that contains only the 15-aa repeats. A representative of Class D (APC-D) corresponds to a fragment that contains only the first 20-aa repeat. A representative of Class E (APC-E) corresponds to a fragment that contains the 20-aa and SAMP repeats. A representative of Class F (APC-F) corresponds to a nonfunctional fragment that contains none of the three APC sites included in the model.

Munemitsu et al. [31] transfected SW480 cells with full-length APC or one of 11 different truncated forms of APC (illustrated in Fig. 3). In our model, full-length APC and the 11 truncated forms of the protein can be grouped into six distinctive classes, Classes A–F (Fig. 3). The proteins in each class are functionally equivalent based on the components and interactions of APC included in the model (Fig. 2). For example, Munemitsu et al. [31] considered three forms of APC each containing the following components: 1) a partial or complete set of the 15-aa repeats, 2) all of the 20-aa repeats, and 3) the SAMP repeats of APC. These are the functional sites that we consider to be included in full-length APC (Fig. 2). Therefore, we will use APC-A to represent all three proteins, as we take these forms of APC to be functionally equivalent. Similarly, we will use APC-B to represent two other proteins, which both contain the 15-aa repeats and only the first 20-aa repeat. We take these two forms to be equivalent to APC1338, the truncated protein in SW480 cells. Henceforth, we will use APC-A, APC-B, APC-C, APC-D, APC-E and APC-F to refer to the proteins in Classes A (e.g., full-length APC), B (e.g., APC1338), C, D, E and F (Fig. 3).

Using the model, we investigated the effects of transfection of SW480 cells with APC-A through APC-F. In the model, the endogeneous concentration of APC1338 in an SW480 cell is set at 100 nM. Similarly, the endogeneous concentration of full-length APC in a normal cell is set at 100 nM (Table 1). Because APC1338 does not contain the third 20-aa repeat, nor SAMP repeats, Axin interactions associated with these sites (Fig. 2) are absent in an SW480 cell. In contrast, in a normal cell, all interactions considered in the model are active, except for the low-affinity interaction between APC and 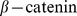 involving the phosphorylated first 20-aa repeat of APC and ARM repeats 3 and 4 of 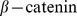. This low-affinity interaction is omitted when considering a normal cell as a simplification (see Materials and Methods). In the model, when a representative of one of the six classes of APC is introduced into an SW480 cell, any novel interactions associated with the functional components of the transfected protein become active. For example, when APC-A is introduced, interactions associated with the third 20-aa repeat and the SAMP repeats (Fig. 2) become active. These interactions are normally missing in an SW480 cell. We assume that simulated transfections each introduce 100 nM of new protein into a cell. Thus, simulated transfection of SW480 with a particular form of APC implies that the cell contains 100 nM of a protein belonging to that form in addition to the 100 nM of the endogeneous form of APC (APC1338). (We systematically investigate how behavior depends on the amount of transfected protein below.)

**Figure 4:**
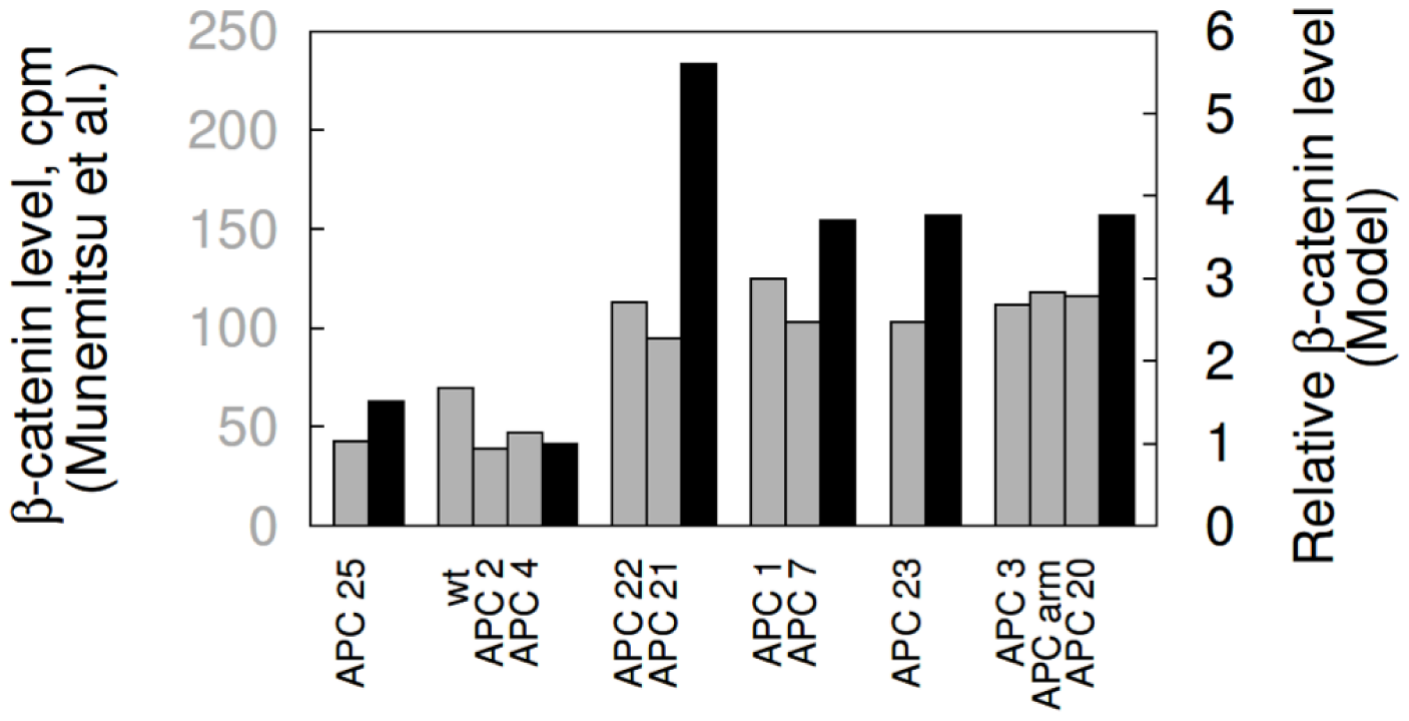
Comparison of simulated and observed effects of transfection of SW480 cells with APC constructs. Relative 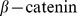 levels in SW480 cells in response to transfection with the APC constructs of Fig. 3 are shown. The gray bars, which correspond to the left 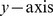, represent experimental data from Munemitsu et al. [31]. The black bars, which correspond to the right 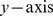, represent model predictions. The predicted concentrations (black bars) are each divided by the concentration of 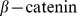 in a normal cell (35 nM, Table 1). For all APC constructs, the same transfection efficiency is assumed. We take a transfected cell to contain 100 nM of added protein. The predicted results therefore represent the effects of 100 nM of a construct in addition to 100 nM of endogeneous APC1338. The simulation results shown here were obtained using BioNetGen input files provided in the Supporting Information: Text S3 was used for the APC-B and APC-F cases, Text S4 was used for the APC-A case, Text S5 was used for the APC-C case, Text S6 was used for the APC-D case, and Text S7 was used for the APC-E case.

In Fig. 4, we compare the model-predicted changes in 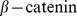 levels in SW480 cells after simulated transfection of different forms of APC against the findings of Munemitsu et al. [31] (Fig. 4). The model is able to recapitulate the qualitative increase or decrease in 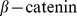 level observed after transfection of each class of protein. Consistent with the findings of Munemitsu et al. [31], the model predicts that only transfection of APC-A and APC-E leads to a decrease in 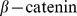 level, whereas the other four classes of APC have the opposite or no effect on 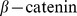 level (Fig. 4) [31]. It should be noted that the results in Fig. 4 were obtained without adjustment or fitting of parameter values.

### Role of full-length APC in *β*–catenin degradation

**Figure 5:**
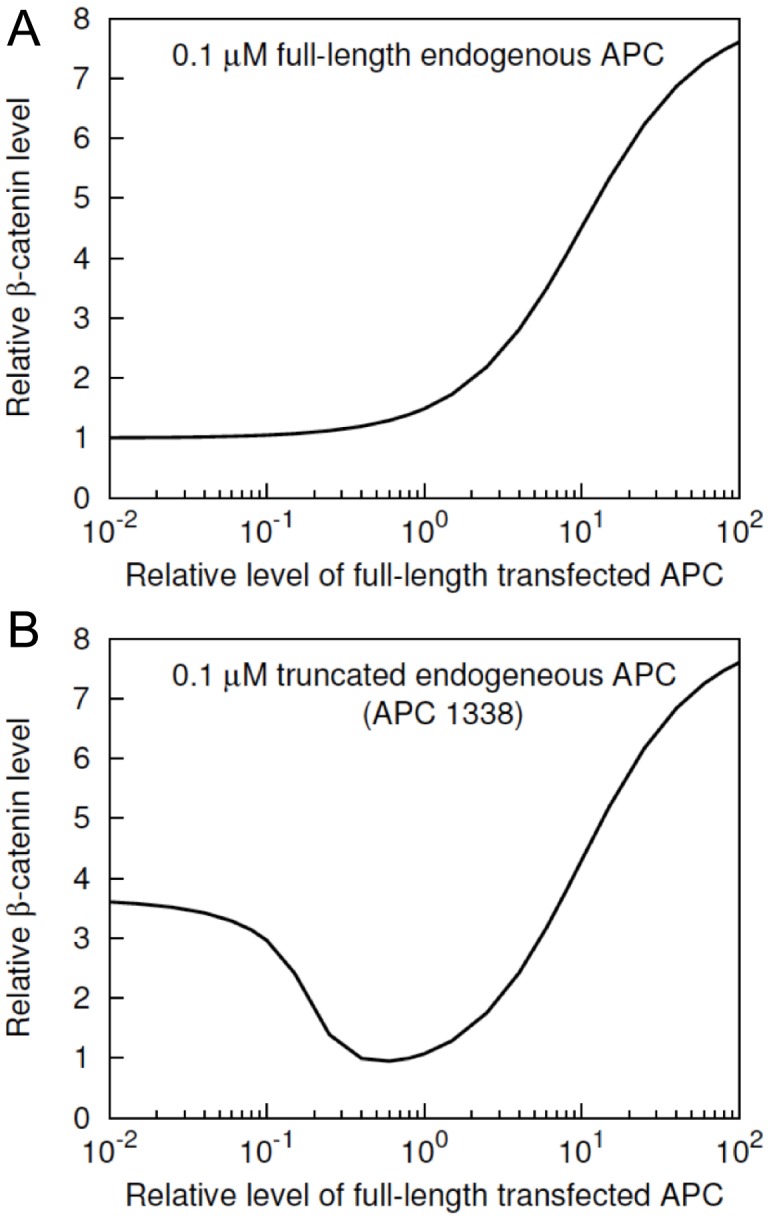
Concentration-dependent effects of full-length APC on 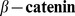. (A) 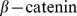 level in a normal cell is shown as a function of APC concentration. The 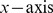 represents the relative amount of APC introduced exogeneously with respect to the endogeneously present 100 nM of full-length APC in a normal cell. The 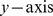 represents the level of 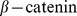 relative to its nominal level in a normal cell (Table 1). (B) 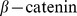 level in an SW480 cell is shown as a function of APC concentration. The 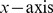 represents the amount of APC introduced exogeneously relative to the endogeneously present 100 nM of APC1338 in an SW480 cell. The 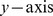 represents the level of 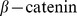 relative to its nominal level in a normal cell, as in panel A. The simulation results shown here were obtained using BioNetGen input files provided in the Supporting Information: Text S2 was used for panel A and Text S4 was used for panel B.

The results of Munemitsu et al. [31] suggest that exogeneous full-length APC downregulates 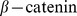 by promoting 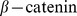 degradation in SW480 cells. Similar results for SW480 cells have been obtained in other studies [33], [34]. However, transfection of different cell types have yielded different results [33]. Using our model, we investigated whether overexpression of APC can generally be expected to increase the rate of 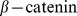 degradation in all cell types, or if the effect may be specific to SW480 cells only (Fig. 5).

Fig. 5A shows the model-predicted 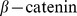 level in a normal cell as a function of APC level. A normal cell in the model is taken to have endogeneous full-length APC at a cytosolic concentration of 100 nM (Table 1). Fig. 5A illustrates the predicted effects of added APC. Fig. 5A shows that increased abundance of APC does not promote 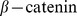 degradation, rather it has a concentration-dependent positive effect on 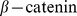 level in normal cells, in contrast to the effect in SW480 cells (Fig. 4). The effects of exogenous full-length APC at different concentrations in SW480 cells are considered in Fig. 5B, which shows the model-predicted 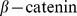 level in SW480 cells as a function of full-length APC level. An SW480 cell is taken to have endogeneous APC1338 at a cytosolic concentration of 100 nM (Table 1). The predicted effect of added full-length APC is a significant decrease in 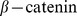 level in SW480 cells over a wide range of exogeneous full-length APC expression levels (Fig. 5B). This finding is consistent with the effects of transient expression of full-length APC in SW480 cells [31] and to some extent also with stable expression of full-length APC in SW480 cells [34].

### Concentration-dependent effects of truncated forms of APC in SW480 cells

**Figure 6:**
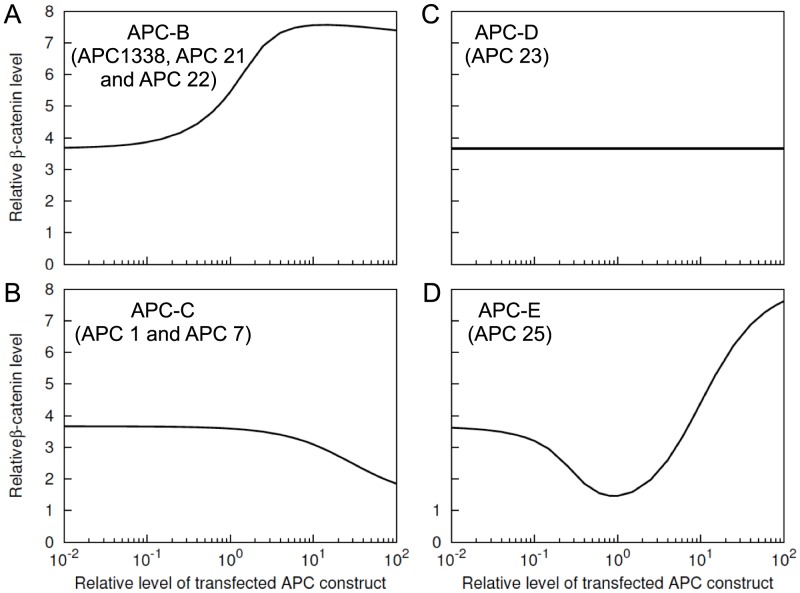
Concentration-dependent effects of APC constructs in SW480 cells. Predicted 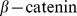 level is shown as a function of expression level for APC-B, -C, -D, and -E. In each panel, the 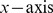 represents the amount of expression relative to the endogeneous level of APC1338 (100 nM). The 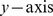 represents the 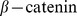 level relative to the nominal level in a normal cell (35 nM, Table 1). Thus, a value of 1 on the 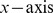 corresponds to a concentration of 100 nM of transfected protein, and a value of 1 on the 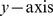 corresponds to a concentration of 35 nM of 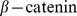. The simulation results shown here were obtained using BioNetGen input files provided in the Supporting Information: Text S3 was used for panel A, Text S5 was used for panel B, Text S6 was used for panel C, and Text S7 was used for panel D.

In Fig. 4, we assumed a fixed amount (100 nM) of exogeneous expression for all six classes of APC. However, the results in Fig. 4 could depend on APC concentration, as seen for APC-A (Fig. 5). Therefore, we investigated the predicted concentration-dependent effects of APC-B, -C, -D and -E on 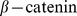 levels in SW480 cells (Fig. 6). For APC-A, such effects have already been discussed (Fig. 5B). We do not consider APC-F because in our model it represents a non-functional form of APC with no binding sites.

The simulation results in Fig. 6 illustrate the concentration-dependent effects of APC-B, -C, -D and -E. As seen in Fig. 6A, added APC-B (e.g., APC1338) increases 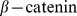 level over the entire concentration range considered. The level of 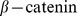 doubles as the amount of exogeneous APC1338 approaches a 10-fold higher amount of endogeneous APC1338 (Fig. 6A). Unlike APC-B, the other three proteins do not increase 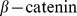 level over the entire concentration range. APC-C reduces 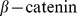 level at relatively high concentrations (Fig. 6B), APC-D does not alter 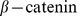 level at any concentration (Fig. 6C), and APC-E reduces 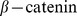 level over a range of intermediate concentrations in a manner similar to full-length APC (cf. Fig. 6D and Fig. 5B).

The only difference between APC-B and APC-C is that the former form of APC contains the first 20-aa repeat, whereas the latter form does not. This distinction leads to APC-B and APC-C having opposite effects on 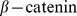 level in SW480 cells (cf. Figs. 6A and 6B). APC-D contains the first 20-aa repeat but no other functional components of APC that are able to interact with 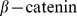 or Axin. Therefore, APC-D cannot interact with 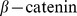 because of the consequent absence of phosphorylation of the 20-aa repeat. The 20-aa repeat in APC-D is never phosphorylated because the unphosphorylated protein is unable to interact with Axin. Thus, APC-D has no effect on 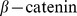 level (Fig. 6C). APC-E entails all structural features of APC-B, but in addition it contains SAMP repeats, which mediate Axin binding (Fig. 2). Because of this distinctive feature, the model predicts that APC-E behaves differently from APC1338 and produces reduced 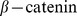 levels at intermediate concentrations of APC-E similar to the predicted effects of full-length APC (Fig. 5B). These results indicate that the absence of SAMP repeats in APC1338 may have an important role in APC1338-mediated increases of 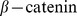 levels in cancer cells.

### Phosphorylation-dependent competition between APC1338 and Axin for binding to *β*–catenin

**Figure 7:**
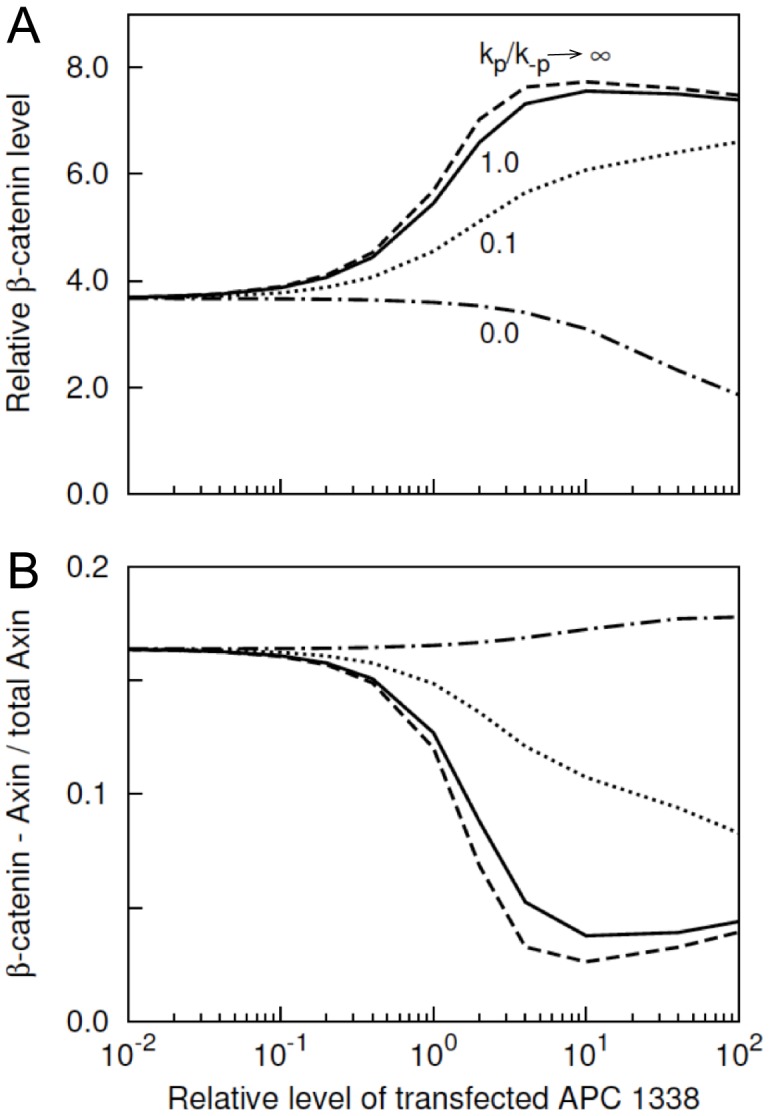
APC1338 phosphorylation and its competition with Axin for 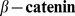. (A) 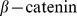 level is shown at different levels of APC1338 phosphorylation. Phosphorylation of APC1338 at the first 20-aa repeat is modulated by changing the values of the phosphorylation and dephosphorylation rate constants 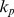 and 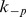. In the figure, the ratio, 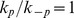 corresponds to the default values of 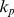 and 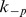 in the model, which are taken to be the same (Table 1). The case where 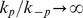 represents an extreme, where the 20-aa repeat always remains phosphorylated. The case where 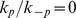 represents the opposite extreme, where APC1338 never becomes phosphorylated. (B) Competition effects on 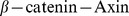 binding arising from APC1338 phosphorylation. The 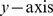 represents the fraction of Axin in complex with 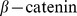. The patterns of the lines represent different phosphorylation and dephosphorylation rate constants, as labeled in panel A. The simulation results shown here were obtained using Text S3, a BioNetGen input file provided in the Supporting Information.

The analysis of Fig. 6 indicated that APC-B (e.g., APC1338) and APC-C have opposite effects on 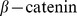 level because APC-B contains a 20-aa repeat that APC-C does not. In Fig. 7, we analyze the effects of phosphorylation of the 20-aa repeat in APC-B on 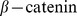 levels in SW480 cells. Recall that phosphorylation of the 20-aa repeat in APC is mediated by 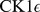 and 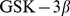 [30], [45] and that phosphorylation of this site is necessary for direct interaction of APC with 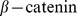 [22], [25].

The simulation results shown in Fig. 7A indicate that phosphorylation of the 20-aa repeat is needed for APC-B/APC1338-mediated stabilization of 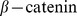. In the figure, the solid line corresponds to default rate constants for phosphorylation and dephosphorylation of APC in the model (Table 1). For these parameter values, the 20-aa repeat is nearly always phosphorylated. This case can be viewed as the extreme opposite of the case where the 20-aa repeat is deleted and therefore never present in phosphorylated form. When the 20-aa repeat is deleted, APC-B becomes equivalent to APC-C and downregulates 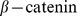 in a similar manner (cf. Fig. 7A and Fig. 6C).

Phosphorylated APC1338 binds to ARM repeats 3 and 4 in 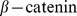, which is also a binding site for Axin (Fig. 2). Thus, phosphorylated APC1338 competes with Axin for binding to 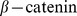 and can inhibit phosphorylation of 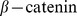 by sequestering 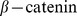 away from Axin-associated kinases. Fig. 7B illustrates the predicted effect of APC1338 phosphorylation on association of 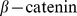 and Axin. The simulation results of Fig. 7B show that phosphorylation of APC1338 inhibits interaction of 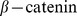 with Axin.

### Mechanism of *β*–catenin upregulation by APC1338

**Figure 8:**
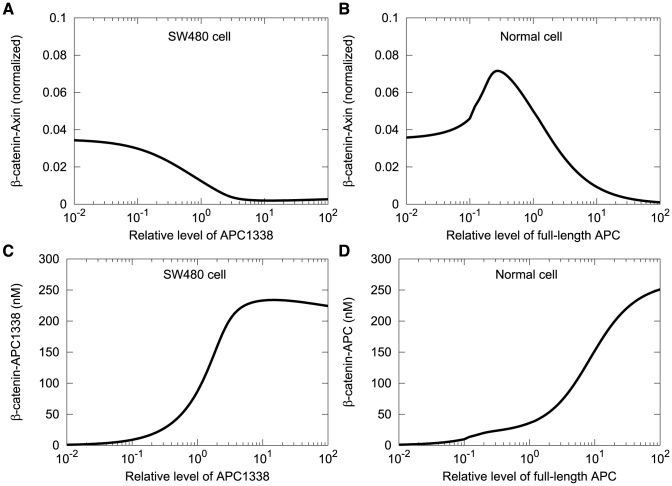
Sequestration of 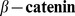 away from Axin by APC1338. (A) Predicted amount of 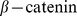 associated either directly or indirectly with Axin is shown as a function of APC1338 concentration in the background of an SW480 cell. The horizontal axis indicates the amount of APC1338 divided by the nominal amount of APC1338 in an SW480 cell (100 nM). The vertical axis indicates the amount of Axin-associated 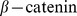 divided by the total amount of 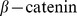 at steady state, which is a function of APC1338 concentration. (B) Predicted amount of 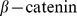 associated either directly or indirectly with Axin is shown as a function of full-length APC concentration in the background of a normal cell. The horizontal axis indicates the amount of full-length APC divided by the nominal amount of full-length APC in a normal cell (100 nM). The vertical axis indicates the amount of Axin-associated 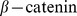 divided by the total amount of 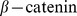 at steady state, which is a function of full-length APC concentration. (C) Predicted amount of 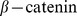 associated directly with APC1338 as a function of relative APC1338 concentration. (D) Predicted amount of 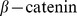 associated directly with full-length APC as a function of relative full-length APC concentration. All results shown were obtained using the parameter values of Table 1, except as indicated. The following BioNetGen input files were used to obtain simulation results: Text S3 was used for panels A and C and Text S2 was used for panels B and D.

The results of Fig. 7 suggest that competition between APC1338 and Axin for 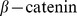 binding upregulates 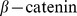 levels in SW480 cells. These results however do not explain how APC1338 and APC regulate 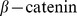 differentially. Differential regulation is somewhat paradoxical because both proteins have phosphorylation sites in the 20-aa repeat region, which mediates 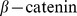 binding. The distinction between APC and APC1338 can be attributed to the absence of SAMP repeats in APC1338, as explained fully below. In short, APC1338 sequesters 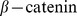 away from Axin, whereas APC fails to do so (Fig. 8). The sequestration effect arises because APC1338, lacking SAMP repeats, cannot mediate indirect association of 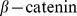 with Axin. We note that the bell-shaped curve in Fig. 8B represents a characteristic scaffold effect [49], [50]. Here, the scaffold is APC and the scaffold ligands are Axin and 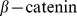.

In normal cells, 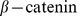 can associate with Axin in two ways: 1) direct binding via ARM repeats 3 and 4 in 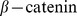 (Arrow 3; Fig. 2), and 2) indirect binding via APC, with APC acting as a linker between 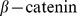 and Axin (Arrows 2 and 4; Fig. 2). As in an SW480 cell, the direct interaction in a normal cell is also inhibited by phosphorylation of the 20-aa repeat region in APC because of competition between phosphorylated APC and Axin for binding to ARM repeats 3 and 4 in 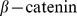. Nonetheless, in a normal cell, the indirect interaction still enables 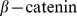 to colocalize with Axin via APC [30], thus allowing phosphorylation of 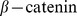 to occur via Axin-associated kinases, which leads to degradation of 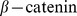. In contrast, in SW480 cells, 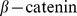 can associate with Axin only through direct interaction. The indirect interaction does not occur because APC1338 lacks the SAMP repeats necessary for Axin binding. Thus, in SW480 cells, APC1338 phosphorylation effectively blocks 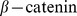 association with Axin, leading to less degradation. A corollary of this finding is that increased expression of Axin would be expected to increase the degradation of 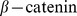, which has been observed [36], [51], [52].

### Effect of stability of the core destruction complex on *β*–catenin level

**Figure 9:**
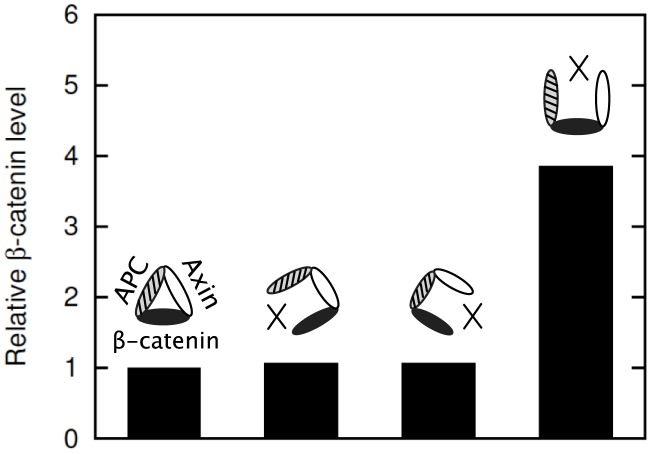
Effects of simulated perturbations of the putative closed/cyclic core destruction complex. Three cases are considered, as indicated in the descriptions of Text S8, S9, S10 (Supporting Information). Each of the three protein-protein interfaces, 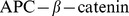 (Text S8), 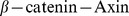 (Text S9), and APC-Axin (Text S10), is ablated such that a closed/cylic structure cannot form. The relative 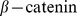 level in each case is compared with the nominal 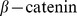 level in a normal cell.

The stability of the destruction complex can be perturbed (decreased) by preventing APC, Axin, and 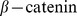 from forming a closed/cyclic ternary complex. The cyclic complex, which we assume can form in normal cells, cannot form in SW480 cells as a result of APC truncation. Formation of the cyclic ternary complex can be prevented not only by truncation of APC but also by other mutations. Any mutation affecting one of the three protein-protein interfaces of the ternary complex would prevent closure of the cyclic structure. Using our model, we simulated inhibition of formation of the cyclic structure by systematically blocking each of the three protein-protein interfaces of the closed/cyclic ternary complex, and we determined the resulting effect on 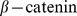 level. As seen in Fig. 9, blocking the contact between APC and 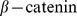 or 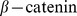 and Axin did not change 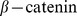 level, indicating that the cyclic structure is unimportant for regulation of 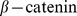 level. Only blocking of the interface between APC and Axin (by removal of SAMP repeats) is predicted to upregulate 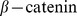 level. However, as established above, this behavior arises for reasons other than destablilization of the cyclic ternary complex of APC, Axin, and 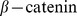. Thus, our model indicates that destabilization of this complex (through ablation of cyclization) is not an important effect of APC truncation.

## Discussion

In this study, we have modeled 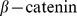 regulation by the destruction complex in normal and colorectal cancer cells, which express full-length and truncated APC, respectively. Our model, illustrated in Figs. 1 and 2, incorporates site-specific mechanistic details about the destruction complex, which comprises a number of signaling proteins. In colorectal cancer cells (e.g., SW480 cells), the interactions of these proteins are altered by truncation of APC. We have used our model to study the function of APC and the effects of its truncation on 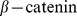 phosphorylation and phosphorylation-dependent degradation. We caution that our results pertain to only the function of APC within an idealized destruction complex and furthermore that we considered the interactions of particular (multifunctional) proteins in isolation from most of their binding partners. Thus, within the context of a cell, the functional effects of APC or truncated APC overexpression could potentially be very different from what our model predicts. Nevertheless, the model is qualitatively consistent with the observed effects of transient expression of various recombinant forms of APC in SW480 cells [31] (Fig. 4). Stronger, less ambiguous tests of model predictions in the future would ideally be performed using an *in vitro* reconstituted or cell-free system [53] to eliminate the uncertainties and complexities of the cellular milieu.

Our analyses indicate that whilst the expression of full-length APC in SW480 cells can be expected to increase degradation of 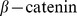, APC overexpression in normal cells may decrease 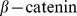 degradation or have no effect. We show that phosphorylation of the first 20-aa repeat in truncated APC, together with the absence of the SAMP repeats, is crucial for the effect of APC1338 on 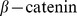 levels in SW480 cells (Figs. 7 and 8). We suggest that phosphorylated APC1338 sequesters 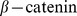 from Axin, thus blocking 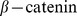 phosphorylation by Axin-bound kinases, viz. 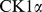 and 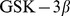. In contrast, phosphorylation of full-length APC, because of its SAMP repeats, which provide an indirect means for interaction between Axin and 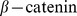, does not block 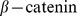 association with Axin and Axin-bound kinases, except at significantly higher levels of expression (cf. panels A and B in Fig. 8).

Several experimental studies have detected competition between phosphorylated full-length APC and Axin for 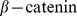 binding [24], [30], [54], [55], although the effect of such competition on 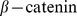 levels has not been previously characterized.

Our results suggest that APC1338, similar to full-length APC, can efficiently mediate competition with Axin for 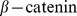 binding, even though it lacks the third 20-aa repeat (Fig. 3), the high-affinity 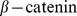 binding site in full-length APC. In the model, APC1338 associates with 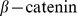 with sufficient strength to displace Axin because of two-point attachment via its 15-aa and phosphorylated 20-aa repeat sites (i.e., because of the combined action of the interactions represented by Arrows 1 and 2 in Figs. 1 and 2). The single-site 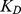
's for the interactions mediated by these sites are 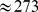 and 80 nM, respectively [25], [43]. The affinities are comparable to the affinity of Axin for 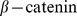 ARM repeats 3 and 4 (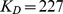 nM [43]). However, if two-point attachment is possible, as we have postulated in our model, then there is an avidity effect. This effect has been studied in other systems [56]–[58] and may confer on phosphorylated APC1338 a competitive advantage, allowing it to outcompete Axin for 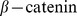.

A critically important feature of our model is a greater abundance of APC than Axin (Table 1). According to our model, as discussed above, truncated APC in SW480 cells acts as a diversion sink that sequesters 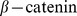 away from Axin. This diversion-sink mechanism cannot be operative if Axin is more abundant than APC. Recent measurements of APC and Axin in SW480 cells indicate that the total amounts of Axin and APC are comparable [59]. At first, these results might seem to contradict the model presented here, which takes APC to be 10-fold more abundant than Axin. However, Axin is not homogeneously distributed in a cell. Much of the Axin in a cell is found in cytoplasmic puncta [35], [36]. Thus, only a fraction of total Axin may be available in a form capable of joining a destruction complex having the composition and structure considered here. A more complicated model than that presented here would be required to account for subcellular compartmentalization of APC, Axin and 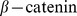, which clearly play an important role in 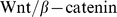 signaling [2], [3]. Such an effort is beyond the intended scope of our study.

The role of colocalization of signaling proteins within the destruction complex is not completely understood. In our model, we assumed that the core of the destruction complex, formed by mutual interactions of APC, Axin, and 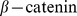, has a closed/cyclic structure (as depicted in the cartoon diagram at the far left of Fig. 9). Within this cyclic structure, there are three protein-protein interfaces, and each of the three interfaces involves interaction between two adjacent proteins, which are connected indirectly via the third protein. Therefore, the binding sites at each interface are confined together in a volume that is small relative to the total volume of the cytoplasm and the local concentrations of tethered binding partners are high. Such high local concentrations can confer on a cyclic structure more stablity than a linear structure of the same composition [60]. It has been assumed that the destruction complex provides a stable platform for phosphorylation of 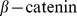 by the Axin-recruited kinases 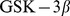 and 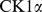. However, according to our analyses, stability of the core destruction complex (i.e., the cyclic ternary complex of APC, Axin and 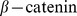) is not important for efficient degradation of 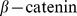. By systematically simulating ablation of each possible contact between 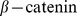, APC, and Axin, we demonstrate that stability of the complex has little if any influence on 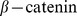 degradation (Fig. 9). (Note that the bar at the far right of Fig. 9 is explained by the diversion-sink mechanism.) We caution that, in the model, stability of the cyclic structure is also determined by factors other than the local concentration effect. Degradation of 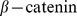 in a core complex can terminate its cyclic structure, leaving behind a complex of APC and Axin only. In addition, phosphorylated APC can disrupt the cyclic structure by breaking the 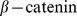-Axin interface through competitive binding and sequestering of 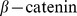 away from the complex.

Questions may arise as to what other roles APC plays besides destruction complex-mediated regulation of 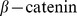 because our model indicates that elevated expression of APC in a normal cell does not have a positive effect on 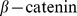 degradation (Fig. 5A), i.e., an increase in APC abundance is not predicted to cause a decrease in 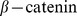 level. A variety of other potential functions of APC have been suggested. Phosphorylated APC has been implicated in subcellular localization and nuclear shuttling of 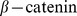 [32], [61]–[63], and high-affinity binding of phosphorylated APC with 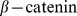 has been suggested to disrupt 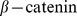 interaction with other binding partners, such as E-cadherin and the Tcf and Lef family transcription factors [24]. It has been shown that APC competes with E-cadherin for binding to the ARM repeat region of 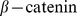 [64]. Indeed, the main effect of stable expression of full-length APC in SW480 cells is not a reduction of 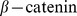 level (although there is an approximate 2-fold reduction in the total amount of 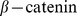), but rather a redistribution of 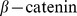 from the nuclear and cytosolic compartments to the plasma membrane [34]. Transient expression of APC (at higher levels) causes a more dramatic reduction in the level of 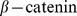 [31], [34]. In future work, it would be interesting to investigate how E-cadherin may regulate 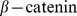 and *vice versa* [3].

Our study identifies 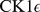 as a potential target for therapeutic intervention in colorectal cancer. Inhibiting 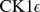 is expected to reverse the effect of truncation of APC in SW480 cells. According to the model, phosphorylation of APC1338 at the first 20-aa repeat plays a key role in upregulating 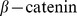 in cancer cells (Fig. 7). Therefore, inhibition of phosphorylation of APC at this site might be an effective way to normalize 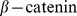 levels in cancer cells. Phosphorylation of APC requires the combined action of two kinases, 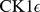 and 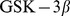 [30]. Therefore, blocking of either kinase is predicted to reduce APC phosphorylation, as shown by Ha et al. [30]. Because 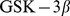 is a common kinase for both 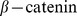 and APC (Fig. 1) and its inhibition would stabilize 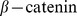, only 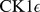 is a potential target. We note that targeting of 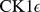 should be feasible in preclinical studies, as pharamacological kinase inhibitors specific to 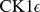 are available [65]–[67].

In this study, we used a detailed mechanistic modeling approach based on the principles of chemical kinetics to investigate regulation of 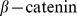 phosphorylation and degradation by full-length and truncated APC. In a previous study, Lee et al. [53] developed a related model to investigate regulation of 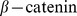 by Wnt stimulation. However, this model does not consider truncated APC. Another notable difference is that the model of Lee et al. [53] is an ordinary differential equation (ODE)-based model, wherein molecules and complexes of signaling proteins are treated as reactive chemical species, which must be enumerated along with all possible reactions to obtain an executable model. In contrast, because of the goals of our study, we developed our model using the rule-based modeling approach [37]–[40]. With this approach, local rules are used to represent protein-protein interactions, which are assumed to be modular. Assumptions of modularity can greatly reduce the complexity of a model for protein-protein interactions, and as a result, enable explicit consideration of multiple functional components within proteins (e.g., the multiple sites of phosphorylation in 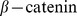). In our model, the components of the proteins considered are the basic reactive elements, i.e., we consider biochemical reactions, such as reversible binding and phosphorylation, to occur at the level of protein sites. This approach was critical for the goals of our study, which included a characterization of the effects of loss of sites in APC. Such effects, and biomolecular site dynamics in general, are difficult to capture in an ODE model [40]. The study presented here provides an example of how rule-based modeling, a fairly new approach in biology, can be used to study biomolecular site dynamics.

We focused on a part of the Wnt/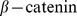 signaling pathway that controls 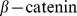 degradation and expression level. Our primary goal was to understand the differential regulation of 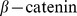 in normal and cancer cells at steady state and in the absence of Wnt signals, unlike in other modeling studies that have focused on the dynamics of 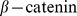 regulation in response to a Wnt ligand [20], [53]. In future work, it would be interesting to extend our model by connecting it to other components of the Wnt/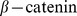 signaling pathway and to further investigate the dynamics of regulation of 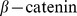.

## Materials and Methods

### Parameter values

Model parameter values are listed in Table 1. Most of the parameter values are based on previously reported estimates. However, some parameter values were set to allow the model to capture a set of observed system behaviors.

In the model, the concentration of 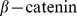 depends in part on its rates of synthesis and degradation. As discussed below, we set parameters for these and other processes considered in the model such that the nominal, steady-state concentration of 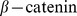 is 35 nM [53], which corresponds to 11,000 copies/cell assuming a cytoplasmic volume of 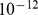 L [59]. This concentration is consistent with the concentration of 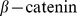 measured in *Xenopus* egg extract [53]. It is also consistent with the cytosolic (but not total) concentration of 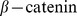 measured in HEK293T and MDCK cells, kidney epithelial cell lines, and in Caco-2 cells [59], an intestinal cell line. We take both APC and 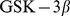 concentration to be 100 nM (31,540 copies/cell). Concentrations of APC and 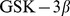 have been measured to be 100 nM and 50 nM, respectively, in *Xenopus* egg extract [53], and measured concentrations of these proteins in mammalian cells fall in the ranges of 4–34 nM and 10–120 nM, respectively [59]. We take 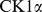 concentration to be the same as 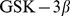, 100 nM. We assume Axin to be present at 10 nM (3,154 copy/cell), which is consistent with recent measurements of Axin abundance in mammalian cells; the Axin concentration measured in mammalian cells ranges from 20 to 150 nM [59]. Lee et al. [53] reported that Axin is present in *Xenopus* egg extract at a very low concentration, in the range of 10 to 20 pM [53]. We rejected this value, while accepting and using the qualitative observation of Lee et al. [53] that Axin is less abundant than APC, because a concentration of 20 pM corresponds to only six copies of Axin per cell for a human epithelial cell, which as stated above is taken to have a cytoplasmic volume of 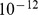 L [59].

For association of any two proteins that are not already in a complex together we assume the same forward rate constant (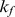) for all interactions: 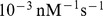 (Table 1). Each reverse rate constant (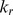) is determined from the relation 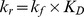, where 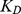 is the equilibrium dissociation constant for the reaction of interest. Equilibrium dissociation constants are set at values reported earlier in the literature, as indicated below.

In the model, the region in 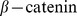 containing ARM repeats 5–9 interacts with the 15-aa repeat region in APC (Arrow 1) with 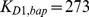 nM (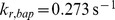) [43]. The region in 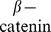 containing ARM repeats 3 and 4 interacts with the phosphorylated 20-aa region in APC (Arrow 2) with an affinity that depends on whether APC is full length or truncated (APC1338). Phosphorylated full-length APC binds the 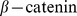 ARM repeats 3 and 4 with 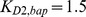 nM (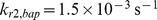), whereas phosphorylated APC1338 binds with 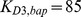 nM (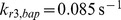) [25]. We assume that APC binds 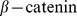 via only a single 20-aa repeat, which is consistent with binding of 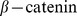 at one 20-aa repeat sterically hindering binding of additional copies of 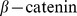 at other 20-aa repeats. When phosphorylated, the third 20-aa repeat mediates binding with 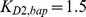 nM, whereas the other six 20-aa repeats appear to bind with much lower affinites [25]. 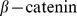 binds the central region of Axin via ARM repeats 3 and 4 (Arrow 3) with 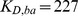 nM (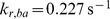) [43]. 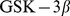 binds the Axin GID domain (Arrow 5) with 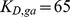 nM (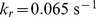) [68]. We assume that APC and 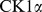 bind Axin (Arrow 4 and 6, respectively) with the same 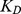: 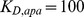 nM (
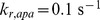
) and 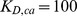 nM (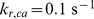).

In the model, 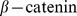 level is determined not only by 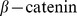 synthesis and degradation rate constants, but also by other parameters that affect the phosphorylation of 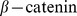 and APC. These parameters include phosphorylation and dephosphorylation rate constants and the enhancement factor 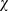. All of these parameters directly or indirectly determine the total level of 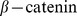. We selected values for these parameters, which are given in Table 1, such that the model captures a set of experimentally-observed system behaviors.

With the parameter values listed in Table 1, the model reproduces a steady-state concentration of (cytosolic) 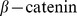 of 35 nM, which as discussed above is consistent with some measurements [53], [59]. Furthermore, the model predicts the effective half-life of 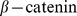 to be 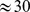 min, and the half-life of 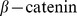 mutated at S33/S37 to be 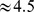 h, which is consistent with the observations of Rubinfeld et al. [14] (Fig. S1). The model also reproduces the experimentally-determined kinetics of 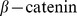 dephosphorylation at distinct phosphorylation sites in response to treatment with LiCl, an inhibitor of 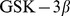 [12] (Fig. S2). The consistency of the model with observed system behaviors is further discussed in the Results section.

We note that the model is not entirely consistent with recent measurements of APC, Axin, (cytosolic) 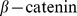, and 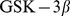 concentrations in SW480 cells [59]. The model cannot simultaneously reproduce these concentrations and observed system behaviors. This could be because the measured concentrations do not reflect the subcellular concentrations relevant for destruction complex function. Alternatively, our mechanistic knowledge of destruction complex function could be incomplete. We formulated our model to probe the limits of understanding of destruction complex function. In setting parameter values, we put an emphasis on selecting values that allow the model to reproduce observed system behaviors, rather than measured system parameters (viz., protein concentrations). This approach is guided by findings from computational analyses of model sloppiness, which indicate that fitting tends to improve the accuracy of model predictions more than parameter measurement [69].

### Model specification

We will refer to the model illustrated in Figs. 1 and 2 as the base model. The base model corresponds to the case of a normal cell with full-length APC. Variants of the base model correspond to SW480 cells with APC1338, SW480 cells transfected with different forms of APC, and other cases considered in this study (see below for further details). The base model and each of its variants was formulated using BNGL, a model-specification language [39]. Executable model-specification files are provided in the Supporting Information for the base model (Text S2) and its variants (Text S3, S4, S5, S6, S7, S8, S9, S10). BNGL is compatible with BioNetGen [39], [70], a software tool for rule-based modeling, and a number of other tools, such as RuleBender [71], an interface for BioNetGen. In addition to parameter values and initial data, each model-specification included molecule type definitions and rules. Molecule type definitions delimit the functional components of proteins and their possible phosphorylation states. The rules characterize protein-protein interactions and other processes (viz., synthesis and degradation of 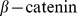). The rules that characterize protein-protein interactions can be subdivided into 10 sets, with one set for each arrow in Fig. 1 or 2. An arrow corresponds to multiple rules if the interaction represented by the rule can take place in multiple contexts and the context influences the rate of reaction. For example, Arrow 3 in Fig. 1 or 2 represents an interaction that can take place between either unconnected proteins or tethered proteins. Thus, there is a rule for each of these two cases.

### Simulation

The base model (Figs. 1 and 2) and each variant model was simulated by submitting the corresponding model-specficiation file to BioNetGen [39], [70] for processing. Model-specification files are provided in the Supporting Information (Text S2, S3, S4, S5, S6, S7, S8, S9, S10); the format of these files is plain text. Simulation protocols are included in each model-specification file. The captions of figures indicate which model-specification files were used in calculations. In all cases, the method used for simulation was an indirect method, meaning that the rules of the model being simulated were not used directly in the simulation procedure. Rather, the rules were expanded (i.e., used to exhaustively enumerate the distinct chemical species and individual chemical reactions implied by the rules) by invoking the generate_network function of BioNetGen to obtain a reaction network. The corresponding system of ODEs describing the mass-action kinetics of this network were then numerically integrated by invoking the simulate_ode function of BioNetGen. BioNetGen uses CVODE [72], [73] for numerical integration of ODEs. For the base model, the reaction network obtained by expansion of its rules comprises 410 distinct chemical species. The size of this network does not reflect the intrinsic complexity of the base model. Rather, the intrinsic complexity of this model is reflected by the number of its rules. The base model includes 29 rules. We used scripts to systematically vary default parameter values specified in BioNetGen input files to produce many of the figures shown in the Results section. Parameter scans are enabled by a function available within RuleBender [71].

## Supporting Information

Figure S1
**The model reproduces the half-lives of** 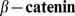 **and** 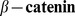 **mutated at S33/S37.** The model predicts the half-life of 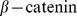 in a normal cell to be 30 min (solid line) and the half-life of 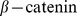 mutated at the S33/S37 phosphorylation site to be 4.5 h (dashed line). These predicted values are consistent with the measured values reported by Rubinfeld et al. [14]. The figure illustrates model-predicted decay of cellular 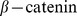 or 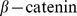 mutated at S33/S37 in a simulated pulse-chase experiment [14]. The model was used to simulate the pulse-chase experiments of Rubenfield et al. [14], which were carried out to determine the half-lives of the proteins.(EPS)

Figure S2
**The model reproduces the effects of LiCl treatment on phosphorylation of cellular** 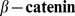 **.** The figure shows predicted and measured phosphorylation of 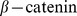 at S45 and S33/S37 in response to LiCl treatment of cells. The solid lines represent model predictions, and the points represent experimental data of Liu et al. [12]. The solid line/filled circles correspond to S45, and the dashed line/open circles correspond to S33/S37. In the model, LiCl addition is simulated by assuming a 20-fold decrease in kinase activity of 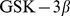 (i.e., 20-fold reduced rate of phosphorylation of the APC 20-aa repeats and S33/S37 in 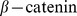). The kinase activities of 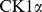 and 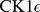 are assumed to be unaffected by LiCl treatment. Time 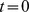 represents the time of LiCl addition. The 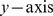 values represent phosphorylation of S45 or S33/S37 relative to the steady-state levels of phosphorylation of these sites in the absence of LiCl.(EPS)

Table S1
**Local parameter sensitivity analysis.** The steady-state level of 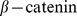 is insensitive to variations of model parameter values, as assessed by local sensitivity coefficients.(PDF)

Text S1 **Model annotation wiki.** This TiddlyWiki (tiddlywiki.com), which takes the form of a single HTML file, provides extensive annotation of the molecule type definitions and rules that comprise the base model, as well as discussion of various modeling assumptions. The wiki can be viewed and navigated using a Web browser. It contains links to relevant information available in public online resources, including UniProt [75], OMIM [76], and Pfam [77].
(TXT)

Text S2
**BioNetGen input file for model illustrated in** **Figs. 1** **and** **2** **.** This file, which can be processed by BioNetGen (after changing the file name extension to .bngl), provides a complete executable specification of the base model 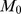, i.e., the model for a normal cell with full-length APC. In this model, the low-affinity interaction of 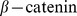 with the first (phosphorylated) 20-aa repeat in APC (Arrow 2 in Figs. 1 and 2) is omitted for simplicity. This simplification does not prevent interaction of 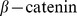 with phosphorylated APC, because a rule is included for the (dominant) high-affinity interaction of 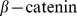 with the third (phosphorylated) 20-aa repeat (Arrow 2 in Figs. 1 and 2). Note that the base model and all of its variants include rules not only for the interactions illustrated in Figs. 1 and 2 but also for synthesis and degradation of 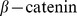 as well as rules for dephosphorylation of APC and 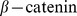. We note that this file can be viewed as specifying a model for a normal cell transfected with APC-A (full-length APC, APC2 or APC 4) (Fig. 3). All forms of APC-A are taken to be functionally equivalent to full-length APC, because each form contains the same functional components that are considered in the base model for full-length APC (15-aa repeats, 20-aa repeats, and SAMP repeats). In simulated transfections of APC-A, the amount of added APC-A (taken to be equivalent to an increase in full-length APC) was varied systematically. See the Simulation subsection in the Materials and Methods section for more information.(TXT)

Text S3
**BioNetGen input file for model variant 1.** This file specifies the model for an SW480 cell with truncated APC (APC1338). This model (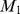) differs from 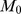 in that full-length APC is replaced by APC1338, meaning that the rule for interaction of Axin with the SAMP repeats in APC is removed (Arrow 4 in Figs. 1 and 2) and the rule for high-affinity interaction of 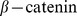 with the third (phosphorylated) 20-aa repeat is replaced by a rule for the low-affinity interaction of 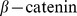 with the first (phosphorylated) 20-aa repeat (Arrow 2 in Figs. 1 and 2). Other interactions involving APC remain the same. These changes are intended to capture the effects of C-terminal truncation of APC, which commonly and in the case of APC1338 means a loss of the SAMP repeats and all but the first 20-aa repeat. We note that this file can also be viewed as specifying a model for an SW480 cell transfected with APC-B or APC-F. Recall that the APC-F class of proteins (APC 3, APC arm, and APC 20) do not contain any of the functional components of full-length APC considered in the model (Fig. 3). Thus, in the model, APC-F does not participate in any interactions (i.e., there are no rules that involve APC-F). Recall that APC-B includes APC 21 and APC 22 (Fig. 3), both of which are taken to be functionally equivalent to APC1338 because these forms of APC, like APC1338, each includes 15-aa repeats and the first 20-aa repeat. Thus, rules for APC1338 interactions are the same as rules for APC-B interactions. In simulated transfections of APC-B, the amount of added APC-B (taken to be equivalent to an increase in endogenous APC1338) was systematically varied. Finally, we note that this file was used for simulating the effect of varying the rate at which the 20-aa repeat region in APC is phosphorylated. In these simulations, the rate constant for APC phosphorylation (denoted as 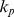 in Table 1 and as kp in model-specification files) was systematically varied.(TXT)

Text S4
**BioNetGen input file for model variant 2.** This file specifies a model for an SW480 cell (i.e., a cell expressing APC1338 endogenously) transfected with APC-A (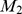). The amount of added APC-A was systematically varied in simulations. Recall that APC-A includes full-length APC, APC 2 and APC 4 (Fig. 3), which are taken to be functionally equivalent because these forms of APC contain 15-aa repeats, all 20-aa repeats, and SAMP repeats. In this model, separate rules are specified for APC1338 and APC-A interactions. The rules account for all of the APC interactions considered in models 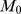 and 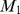.(TXT)

Text S5
**BioNetGen input file for model variant 3.** This file specifies a model for an SW480 cell transfected with APC-C (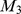). The amount of added APC-C was systematically varied in simulations. Recall that APC-C includes APC 21 and APC 22 (Fig. 3), which are taken to be functionally equivalent because both of these forms of APC contain 15-aa repeats while missing all 20-aa repeats and the SAMP repeats. In this model, separate rules are specified for APC1388 and APC-C interactions. The only interaction in which APC-C can participate is the constitutive interaction between APC and 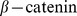 (Arrow 1 in Figs. 1 and 2).(TXT)

Text S6
**BioNetGen input file for model variant 4.** This file specifies a model for an SW480 cell transfected with APC-D (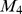). The amount of added APC-D was systematically varied in simulations. Recall that APC-D includes only APC 23 (Fig. 3), which contains only the first 20-aa repeat. Although APC-D contains a 20-aa repeat, APC-D cannot be phosphorylated because it lacks other components needed for association with Axin. Thus, this model is essentially the same as model 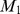.(TXT)

Text S7
**BioNetGen input file for model variant 5.** This file specifies a model for an SW480 cell transfected with APC-E (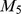). The amount of added APC-E (or APC 25, Fig. 3) was systematically varied in simulations. In this model, separate rules are specified for APC1388 and APC-E interactions. APC-E participates in the same interactions as APC1338 except for the constitutive interaction between APC and 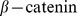 (Arrow 1 in Figs. 1 and 2), which is mediated by the 15-aa repeats that are missing in APC-E.(TXT)

Text S8
**BioNetGen input file for model variant 6.** This model (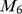) is the same as 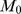 except that the association rate constant for APC binding to 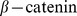 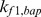 (denoted as kf1_bap in model-specification files) has been set to 0. Note that 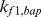 characterizes the interaction represented by Arrow 1 in Figs. 1 and 2.(TXT)

Text S9
**BioNetGen input file for model variant 7.** This model (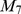) is the same as 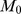 except that the association rate constant for Axin binding to 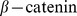 (denoted as 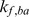 in Table 1 and as kf_ba in model-specification files) has been set to 0. Recall that Arrow 3 in Figs. 1 and 2 represents the interaction between Axin and 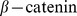.(TXT)

Text S10
**BioNetGen input file for model variant 8.** This model (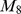) is the same as 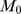 except that the association rate constant for APC binding to Axin (denoted as 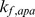 in Table 1 and as kf_apa in model-specification files) has been set to 0. Recall that Arrow 4 in Figs. 1 and 2 represents the interaction between APC and Axin.(TXT)

## References

[1] Clevers H (2006) Wnt/*β*–catenin signaling in development and disease. Cell 127: 469–80. 17081971 10.1016/j.cell.2006.10.018

[2] Nusse R, Varmus H (2012) Three decades of Wnt: a personal perspective on how a scientific field developed. EMBO J 31: 2670–84. 22617420 10.1038/emboj.2012.146 PMC3380217

[3] Burgess A, Faux M, Layton M, Ramsay R (2011) Wnt signaling and colon tumorigenesis — a view from the periphery. Exp Cell Res 317: 2748–58. 21884696 10.1016/j.yexcr.2011.08.010

[4] Behrens J, von Kries JP, Kühl M, Bruhn L, Wedlich D, et al (1996) Functional interaction of *β*–catenin with the transcription factor LEF-1. Nature 382: 638–42. 8757136 10.1038/382638a0

[5] Molenaar M, van de Wetering M, Oosterwegel M, Peterson-Maduro J, Godsave S, et al (1996) XTcf-3 transcription factor mediates *β*–catenin-induced axis formation in Xenopus embryos. Cell 86: 391–9. 8756721 10.1016/s0092-8674(00)80112-9

[6] van de Wetering M, Cavallo R, Dooijes D, van Beest M, van Es J, et al (1997) Armadillo coactivates transcription driven by the product of the Drosophila segment polarity gene dTCF. Cell 88: 789–99. 9118222 10.1016/s0092-8674(00)81925-x

[7] Zhurinsky J, Shtutman M, Ben-Ze'ev A (2000) Differential mechanisms of LEF/TCF family-dependent transcriptional activation by *β*–catenin and plakoglobin. Mol Cell Biol 20: 4238–52. 10825188 10.1128/mcb.20.12.4238-4252.2000 PMC85792

[8] Peifer M, Polakis P (2000) Wnt signaling in oncogenesis and embryogenesis—a look outside the nucleus. Science 287: 1606–9. 10733430 10.1126/science.287.5458.1606

[9] Kimelman D, Xu W (2006) *β*–catenin destruction complex: insights and questions from a structural perspective. Oncogene 25: 7482–91. 17143292 10.1038/sj.onc.1210055

[10] Stamos JL, Weis WI (2013) The *β*–catenin destruction complex. Cold Spring Harb Perspect Biol 5: a007898. 23169527 10.1101/cshperspect.a007898 PMC3579403

[11] Dajani R, Fraser E, Roe SM, Yeo M, Good VM, et al (2003) Structural basis for recruitment of glycogen synthase kinase 3*β* to the axin-APC scaffold complex. EMBO J 22: 494–501. 12554650 10.1093/emboj/cdg068 PMC140752

[12] Liu C, Li Y, Semenov M, Han C, Baeg GH, et al (2002) Control of *β*–catenin phosphorylation/degradation by a dual-kinase mechanism. Cell 108: 837–47. 11955436 10.1016/s0092-8674(02)00685-2

[13] Wu D, Pan W (2010) GSK3: a multifaceted kinase in Wnt signaling. Trends Biochem Sci 35: 161–8. 19884009 10.1016/j.tibs.2009.10.002 PMC2834833

[14] Rubinfeld B, Robbins P, El-Gamil M, Albert I, Porfiri E, et al (1997) Stabilization of *β*–catenin by genetic defects in melanoma cell lines. Science 275: 1790–2. 9065403 10.1126/science.275.5307.1790

[15] Aberle H, Bauer A, Stappert J, Kispert A, Kemler R (1997) *β*–catenin is a target for the ubiquitin-proteasome pathway. EMBO J 16: 3797–804. 9233789 10.1093/emboj/16.13.3797 PMC1170003

[16] Cliffe A, Hamada F, Bienz M (2003) A role of Dishevelled in relocating Axin to the plasma membrane during Wingless signaling. Curr Biol 13: 960–6. 12781135 10.1016/s0960-9822(03)00370-1

[17] Schweizer L, Varmus H (2003) Wnt/Wingless signaling through *β*–catenin requires the function of both LRP/Arrow and frizzled classes of receptors. BMC Cell Biol 4: 4. 12729465 10.1186/1471-2121-4-4 PMC156895

[18] Cong F, Schweizer L, Varmus H (2004) Wnt signals across the plasma membrane to activate the *β*–catenin pathway by forming oligomers containing its receptors, Frizzled and LRP. Development 131: 5103–15. 15459103 10.1242/dev.01318

[19] Li VSW, Ng SS, Boersema PJ, Low TY, Karthaus WR, et al (2012) Wnt signaling through inhibition of *β*–catenin degradation in an intact Axin1 complex. Cell 149: 1245–56. 22682247 10.1016/j.cell.2012.05.002

[20] Hernández AR, Klein AM, Kirschner MW (2012) Kinetic responses of *β*–catenin specify the sites of Wnt control. Science 338: 1337–40. 23138978 10.1126/science.1228734

[21] Markowitz SD, Bertagnolli MM (2009) Molecular basis of colorectal cancer. N Engl J Med 361: 2449–60. 20018966 10.1056/NEJMra0804588 PMC2843693

[22] Xing Y, Clements WK, Kimelman D, Xu W (2003) Crystal structure of a *β*–catenin/axin complex suggests a mechanism for the *β*–catenin destruction complex. Genes Dev 17: 2753–64. 14600025 10.1101/gad.1142603 PMC280624

[23] Sakanaka C, Williams LT (1999) Functional domains of axin. Importance of the C terminus as an oligomerization domain. J Biol Chem 274: 14090–3. 10318824 10.1074/jbc.274.20.14090

[24] Xing Y, Clements WK, Le Trong I, Hinds TR, Stenkamp R, et al (2004) Crystal structure of a *β*–catenin/APC complex reveals a critical role for APC phosphorylation in APC function. Mol Cell 15: 523–33. 15327769 10.1016/j.molcel.2004.08.001

[25] Liu J, Xing Y, Hinds TR, Zheng J, Xu W (2006) The third 20 amino acid repeat is the tightest binding site of APC for *β*–catenin. J Mol Biol 360: 133–44. 16753179 10.1016/j.jmb.2006.04.064

[26] Eklof Spink K, Fridman SG, Weis WI (2001) Molecular mechanisms of *β*–catenin recognition by adenomatous polyposis coli revealed by the structure of an APC-*β*–catenin complex. EMBO J 20: 6203–12. 11707392 10.1093/emboj/20.22.6203 PMC125720

[27] Spink KE, Polakis P, Weis WI (2000) Structural basis of the Axin-adenomatous polyposis coli interaction. EMBO J 19: 2270–9. 10811618 10.1093/emboj/19.10.2270 PMC384355

[28] Hedgepeth CM, Deardorff MA, Rankin K, Klein PS (1999) Regulation of glycogen synthase kinase 3*β* and downstream Wnt signaling by axin. Mol Cell Biol 19: 7147–57. 10490650 10.1128/mcb.19.10.7147 PMC84708

[29] Sobrado P, Jedlicki A, Bustos VH, Allende CC, Allende JE (2005) Basic region of residues 228–231 of protein kinase CK1*α* is involved in its interaction with axin: binding to axin does not affect the kinase activity. J Cell Biochem 94: 217–24. 15565646 10.1002/jcb.20350

[30] Ha NC, Tonozuka T, Stamos JL, Choi HJ, Weis WI (2004) Mechanism of phosphorylation-dependent binding of APC to *β*–catenin and its role in *β*–catenin degradation. Mol Cell 15: 511–21. 15327768 10.1016/j.molcel.2004.08.010

[31] Munemitsu S, Albert I, Souza B, Rubinfeld B, Polakis P (1995) Regulation of intracellular *β*–catenin levels by the adenomatous polyposis coli (APC) tumor-suppressor protein. Proc Natl Acad Sci U S A 92: 3046–50. 7708772 10.1073/pnas.92.7.3046 PMC42356

[32] Seo E, Jho Eh (2007) Axin-independent phosphorylation of APC controls *β*–catenin signaling via cytoplasmic retention of *β*–catenin. Biochem Biophys Res Commun 357: 81–6. 17418091 10.1016/j.bbrc.2007.03.117

[33] Yang J, Zhang W, Evans PM, Chen X, He X, et al (2006) Adenomatous polyposis coli (APC) differentially regulates *β*–catenin phosphorylation and ubiquitination in colon cancer cells. J Biol Chem 281: 17751–7. 16798748 10.1074/jbc.M600831200

[34] Faux MC, Ross JL, Meeker C, Johns T, Ji H, et al (2004) Restoration of full-length adenomatous polyposis coli (APC) protein in a colon cancer cell line enhances cell adhesion. J Cell Sci 117: 427–39. 14679305 10.1242/jcs.00862

[35] Schwarz-Romond T, Metcalfe C, Bienz M (2007) Dynamic recruitment of axin by Dishevelled protein assemblies. J Cell Sci 120: 2402–12. 17606995 10.1242/jcs.002956

[36] Faux M, Coates J, Catimel B, Cody S, Clayton A, et al (2008) Recruitment of ademonatous polyposis coli and *β*–catenin to axin-puncta. Oncogene 27: 5808–20. 18591934 10.1038/onc.2008.205

[37] Hlavacek WS, Faeder JR, Blinov ML, Posner RG, Hucka M, et al (2006) Rules for modeling signal-transduction systems. Sci STKE 2006: re6. 16849649 10.1126/stke.3442006re6

[38] Faeder JR, Blinov ML, Goldstein B, Hlavacek WS (2005) Rule-based modeling of biochemical networks. Complexity 10: 22–41.

[39] Faeder JR, Blinov ML, Hlavacek WS (2009) Rule-based modeling of biochemical systems with BioNetGen. Methods Mol Biol 500: 113–67. 19399430 10.1007/978-1-59745-525-1_5

[40] Chylek LA, Stites EC, Posner RG, Hlavacek WS (2013) Innovations of the rule-based modeling approach. In: Prokop A, Csukás B, Editors. Systems Biology: Integrative Biology and Simulation Tools, Volume 1. Springer.

[41] Hlavacek WS, Faeder JR, Blinov ML, Perelson AS, Goldstein B (2003) The complexity of complexes in signal transduction. Biotechnol Bioeng 84: 783–94. 14708119 10.1002/bit.10842

[42] Chylek LA, Hu B, Blinov ML, Emonet T, Faeder JR, et al (2011) Guidelines for visualizing and annotating rule-based models. Mol BioSyst 7: 2779–95. 21647530 10.1039/c1mb05077j PMC3168731

[43] Kishida S, Yamamoto H, Ikeda S, Kishida M, Sakamoto I, et al (1998) Axin, a negative regulator of the wnt signaling pathway, directly interacts with adenomatous polyposis coli and regulates the stabilization of *β*–catenin. J Biol Chem 273: 10823–6. 9556553 10.1074/jbc.273.18.10823

[44] Frame S, Cohen P (2001) GSK3 takes centre stage more than 20 years after its discovery. Biochem J 359: 1–16. 11563964 10.1042/0264-6021:3590001 PMC1222116

[45] Rubinfeld B, Tice DA, Polakis P (2001) Axin-dependent phosphorylation of the adenomatous polyposis coli protein mediated by casein kinase 1*ε* . J Biol Chem 276: 39037–45. 11487578 10.1074/jbc.M105148200

[46] Ikeda S, Kishida M, Matsuura Y, Usui H, Kikuchi A (2000) GSK-3*β*-dependent phosphorylation of adenomatous polyposis coli gene product can be modulated by *β*–catenin and protein phosphatase 2A complexed with Axin. Oncogene 19: 537–45. 10698523 10.1038/sj.onc.1203359

[47] Crothers DM, Metzger H (1972) The influence of polyvalency on the binding properties of antibodies. Immunochemistry 9: 341–57. 4113719 10.1016/0019-2791(72)90097-3

[48] Smith KJ, Johnson KA, Bryan TM, Hill DE, Markowitz S, et al (1993) The APC gene product in normal and tumor cells. Proc Natl Acad Sci U S A 90: 2846–50. 8385345 10.1073/pnas.90.7.2846 PMC46193

[49] Yang J, Hlavacek WS (2011) Scaffold-mediated nucleation of protein signaling complexes: elementary principles. Math Biosci 232: 164–73. 21683720 10.1016/j.mbs.2011.06.003 PMC3137898

[50] Douglass EF Jr, Miller CJ, Sparer G, Shapiro H, Spiegel DA (2013) A comprehensive mathematical model for three-body binding equilibria. J Am Chem Soc 135: 6092–9. 23544844 10.1021/ja311795d PMC3717292

[51] Behrens J, Jerchow BA, Würtele M, Grimm J, Asbrand C, et al (1998) Functional interaction of an axin homolog, conductin, with *β*–catenin, APC, and GSK3*β* . Science 280: 596–9. 9554852 10.1126/science.280.5363.596

[52] Hart MJ, de los Santos R, Albert IN, Rubinfeld B, Polakis P (1998) Downregulation of *β*–catenin by human Axin and its association with the APC tumor suppressor, *β*–catenin and GSK3*β* . Curr Biol 8: 573–81. 9601641 10.1016/s0960-9822(98)70226-x

[53] Lee E, Salic A, Krüger R, Heinrich R, Kirschner MW (2003) The roles of APC and Axin derived from experimental and theoretical analysis of the Wnt pathway. PLoS Biol 1: E10. 14551908 10.1371/journal.pbio.0000010 PMC212691

[54] Graham TA, Weaver C, Mao F, Kimelman D, Xu W (2000) Crystal structure of a *β*–catenin/Tcf complex. Cell 103: 885–96. 11136974 10.1016/s0092-8674(00)00192-6

[55] von Kries JP, Winbeck G, Asbrand C, Schwarz-Romond T, Sochnikova N, et al (2000) Hot spots in *β*–catenin for interactions with LEF-1, conductin and APC. Nat Struct Biol 7: 800–7. 10966653 10.1038/79039

[56] O'Brien R, Rugman P, Renzoni D, Layton M, Handa R, et al (2000) Alternative modes of binding of proteins with tandem SH2 domains. Protein Sci 9: 570–9. 10752619 10.1110/ps.9.3.570 PMC2144564

[57] Barua D, Faeder JR, Haugh JM (2008) Computational models of tandem SRC homology 2 domain interactions and application to phosphoinositide 3-kinase. J Biol Chem 283: 7338–45. 18204097 10.1074/jbc.M708359200 PMC2276335

[58] Kuriyan J, Cowburn D (1997) Modular peptide recognition domains in eukaryotic signaling. Annu Rev Biophys Biomol Struct 26: 259–88. 9241420 10.1146/annurev.biophys.26.1.259

[59] Tan CW, Gardiner BS, Hirokawa Y, Layton MJ, Smith DW, et al (2012) Wnt signalling pathway parameters for mammalian cells. PLOS ONE 7: e31882. 22363759 10.1371/journal.pone.0031882 PMC3283727

[60] Jacobson H, Stockmayer WH (1950) Intramolecular reaction in polycondensations. I. The theory of linear systems. J Chem Phys 18: 1600–6.

[61] Bienz M (2002) The subcellular destinations of APC proteins. Nat Rev Mol Cell Biol 3: 328–38. 11988767 10.1038/nrm806

[62] Henderson BR (2000) Nuclear-cytoplasmic shuttling of APC regulates *β*–catenin subcellular localization and turnover. Nat Cell Biol 2: 653–60. 10980707 10.1038/35023605

[63] Rosin-Arbesfeld R, Townsley F, Bienz M (2000) The APC tumour suppressor has a nuclear export function. Nature 406: 1009–12. 10984057 10.1038/35023016

[64] Hülsken J, Birchmeier W, Behrens J (1994) E-cadherin and APC compete for the interaction with *β*–catenin and the cytoskeleton. J Cell Biol 127: 2061–9. 7806582 10.1083/jcb.127.6.2061 PMC2120290

[65] Behrend L, Milne DM, Stöter M, Deppert W, Campbell LE, et al (2000) IC261, a specific inhibitor of the protein kinases casein kinase 1-delta and -epsilon, triggers the mitotic checkpoint and induces p53-dependent postmitotic effects. Oncogene 19: 5303–13. 11103931 10.1038/sj.onc.1203939

[66] Badura L, Swanson T, Adamowicz W, Adams J, Cianfrogna J, et al (2007) An inhibitor of casein kinase I*ε* induces phase delays in circadian rhythms under free-running and entrained conditions. J Pharmacol Exp Ther 322: 730–8. 17502429 10.1124/jpet.107.122846

[67] Walton K, Fisher K, Rubitski D, Marconi M, Meng Q, et al (2009) Selective inhibition of casein kinase 1*ε* minimally alters circadian clock period. J Pharmacol Exp Ther 330: 430–9. 19458106 10.1124/jpet.109.151415

[68] Ikeda S, Kishida S, Yamamoto H, Murai H, Koyama S, et al (1998) Axin, a negative regulator of the Wnt signaling pathway, forms a complex with GSK-3*β* and *β*–catenin and promotes GSK-3*β*-dependent phosphorylation of *β*–catenin. EMBO J 17: 1371–84. 9482734 10.1093/emboj/17.5.1371 PMC1170485

[69] Gutenkunst RN, Waterfall JJ, Casey FP, Brown KS, Myers CR, et al (2007) Universally sloppy parameter sensitivities in systems biology models. PLoS Comput Biol 3: 1871–8. 17922568 10.1371/journal.pcbi.0030189 PMC2000971

[70] Blinov ML, Faeder JR, Goldstein B, Hlavacek WS (2004) BioNetGen: software for rule-based modeling of signal transduction based on the interactions of molecular domains. Bioinformatics 20: 3289–91. 15217809 10.1093/bioinformatics/bth378

[71] Xu W, Smith AM, Faeder JR, Marai GE (2011) RuleBender: a visual interface for rule-based modeling. Bioinformatics 27: 1721–2. 21493655 10.1093/bioinformatics/btr197 PMC3106190

[72] Cohen S, Hindmarsh A (1996) CVODE, a stiff/nonstiff ODE solver in C. Comp Phys 10: 138–43.

[73] Hindmarsh A, Brown P, Grant K, Lee S, Serban R, et al (2005) SUNDIALS: Suite of nonlinear and differential/algebraic equation solvers. ACM Trans Math Softw 31: 363–96.

[74] Sadot E, Conacci-Sorrell M, Zhurinsky J, Shnizer D, Lando Z, et al (2002) Regulation of S33/S37 phosphorylated *β*–catenin in normal and transformed cells. J Cell Sci 115: 2771–80. 12077367 10.1242/jcs.115.13.2771

[75] Jain E, Bairoch A, Duvaud S, Phan I, Redaschi N, et al (2009) Infrastructure for the life sciences: design and implementation of the UniProt website. BMC Bioinformatics 10: 136. 19426475 10.1186/1471-2105-10-136 PMC2686714

[76] Amberger J, Bocchini C, Hamosh A (2011) A new face and new challenges for Online Medelian Inheritance in Man (OMIM). Hum Mut 32: 564–7. 21472891 10.1002/humu.21466

[77] Finn RD, Mistry J, Tate J, Coggill P, Heger A, et al (2010) The Pfam protein families database. Nucleic Acids Res 38: D211–22. 19920124 10.1093/nar/gkp985 PMC2808889

