# Supplementary material for: Modeling the Effect of APC Truncation on Destruction Complex Function in Colorectal Cancer Cells
Source: PLoS Comput Biol. 2013 Sep 26;9(9):e1003217. doi: 10.1371/journal.pcbi.1003217 (PMC3784502; doi:10.1371/journal.pcbi.1003217)
Supplement: Table S1 — Local parameter sensitivity analysis. The steady-state level of is insensitive to variations of model parameter values, as assessed by local sensitivity coefficients. (PDF) [file pcbi.1003217.s003.pdf]

Table S1: Local parameter sensitivity analysis.

Parameter	Sensitivity coefficient[1]	Comment
$k_{s,b}$	1.02	β-catenin synthesis rate constant
$AXIN_{tot}$	-9.74×10^{-01}	Axin concentration
$k_{p,b}$	-9.22×10^{-01}	β-catenin phosphorylation rate constant
$k_{f,apa}$	-5.07×10^{-01}	Axin binding to the APC SAMP repeats (forward rate constant)
APC_{tot}	4.84×10^{-01}	APC concentration
$k_{r,apa}$	4.60×10^{-01}	Axin binding to the APC SAMP repeats (dissociation rate constant)
$k_{-p,b}$	3.45×10^{-01}	β-catenin dephosphorylation rate constant
$CK1\alpha_{tot}$	-3.00×10^{-01}	CK1α concentration
$k_{f,ca}$	-2.85×10^{-01}	CK1α binding to Axin (forward rate constant)
$k_{r,ca}$	2.59×10^{-01}	CK1α binding to Axin (dissociation rate constant)
GSK_{tot}	-2.32×10^{-01}	GSK-3β concentration
$k_{d,b2}$	-2.24×10^{-01}	β-catenin degradation (fast degradation rate constant)
$k_{f,ga}$	-2.19×10^{-01}	GSK-3β binding to Axin (forward rate constant)
$k_{r,ga}$	2.04×10^{-01}	GSK-3β binding to Axin (dissociation rate constant)
$k_{d,b1}$	-6.56×10^{-02}	β-catenin degradation (slow degradation rate constant)
$k_{f,ba}$	-3.55×10^{-02}	β-catenin ARM repeats 3 and 4 binding to Axin (forward rate constant)
$k_{r,ba}$	2.85×10^{-02}	β-catenin ARM repeats 3 and 4 binding to Axin (dissociation rate constant)
k_p	2.54×10^{-02}	APC phosphorylation rate constant
k_{-p}	-2.50×10^{-02}	APC dephosphorylation rate constant
$k_{f2,bap}$	2.15×10^{-02}	β-catenin ARM repeats 3 and 4 binding to phosphorylated APC 20-aa repeat (foreward rate constant)
$k_{r1,bap}$	1.06×10^{-02}	β-catenin ARM repeats 5-9 binding to APC 15-aa repeat region (dissociation rate constant)
$k_{r2,bap}$	-1.03×10^{-02}	β-catenin ARM repeats 3 and 4 binding to phosphorylated APC 20-aa repeat (dissociation rate constant)
χ	-1.01×10^{-02}	Enhancement factor for intracomplex binding
$k_{f1,bap}$	-7.53×10^{-03}	β-catenin ARM repeats 5-9 binding to APC 15-aa repeat region (forward rate constant)
$BCAT_{tot}$	N/A	β-catenin concentration

[1]In general local (differential) sensitivity coefficients are defined as $\frac{x_i}{y_j}\left(\frac{\partial y_j}{\partial x_i}\right)$, where x_i represents the value of a model parameter and y_j represents the steady-state value of a model variable. Here, we focus on sensitivity coefficients for the steady-state level of β-catenin. The partial derivative appearing in the definition of each sensitivity coefficient is calculated via a finite-difference approximation: $\frac{\partial y_j}{\partial x_i} \sim \frac{\Delta y_j}{\Delta x_i}$, where Δx_i represents a 1% change in the nominal value of parameter x_i (Table 1) and Δy_j represents the resulting change in the steady-state value of the variable y_j (i.e., the steady-state concentration of β-catenin). A positive (negative) sensitivity coefficient indicates that an increase in the value of the corresponding parameter value causes an increase (decrease) in the steady-state value of the corresponding variable (y_j , the steady-state concentration of β-catenin). The sensitivity coefficients given here characterize the robustness of the nominal steady state for a normal cell (i.e., the form of the model used to consider the case of full-length APC).
